# Comparative analysis of the LARP1 C-terminal DM15 region through Coelomate evolution

**DOI:** 10.1371/journal.pone.0308574

**Published:** 2024-08-27

**Authors:** Elaine Nguyen, Jahree A. Sosa, Kevin C. Cassidy, Andrea J. Berman

**Affiliations:** 1 Biological Sciences, University of Pittsburgh, Pittsburgh, PA, United States of America; 2 BIOVIA, Dassault Systèmes, Waltham, MA, United States of America; Universidad de Jaen, SPAIN

## Abstract

TOR (target of rapamycin), a ubiquitous protein kinase central to cellular homeostasis maintenance, fundamentally regulates ribosome biogenesis in part by its target La-related protein 1 (LARP1). Among other target transcripts, LARP1 specifically binds TOP (terminal oligopyrimidine) mRNAs encoding all 80 ribosomal proteins in a TOR-dependent manner through its C-terminal region containing the DM15 module. Though the functional implications of the LARP1 interaction with target mRNAs is controversial, it is clear that the TOP-LARP1-TOR axis is critical to cellular health in humans. Its existence and role in evolutionarily divergent animals remain less understood. We focused our work on expanding our knowledge of the first arm of the axis: the connection between LARP1-DM15 and the 5’ TOP motif. We show that the overall DM15 architecture observed in humans is conserved in fruit fly and zebrafish. Both adopt familiar curved arrangements of HEAT-like repeats that bind 5’ TOP mRNAs on the same conserved surface, although molecular dynamics simulations suggest that the N-terminal fold of the fruit fly DM15 is predicted to be unstable and unfold. We demonstrate that each ortholog interacts with TOP sequences with varying affinities. Importantly, we determine that the ability of the DM15 region to bind some TOP sequences but not others might amount to the context of the RNA structure, rather than the ability of the module to recognize some sequences but not others. We propose that TOP mRNAs may retain similar secondary structures to regulate LARP1 DM15 recognition.

## Introduction

The Target of rapamycin (TOR) is a well conserved serine/threonine kinase that regulates cellular physiology in organisms across all clades of Eukarya [1]. TOR integrates metabolic signals to direct the translational programming of a cell [2]. Among the most sensitive transcripts subject to TOR-dependent regulation in humans are TOP mRNAs, which are characterized by a tract of pyrimidines, the 5’ TOP motif, immediately adjacent the 5’ 7-methylguanosine cap [3–6]. In humans, TOP mRNAs encode all 80 ribosomal protein components, many translation factors, and several RNA-binding proteins, among other protein classes [7–9]. Regulation of TOP mRNA translation is therefore critical to tuning ribosome biogenesis.

Though the mTOR complex 1 (mTORC1) controls the translation of TOP mRNAs in mammals, it requires a transducer RNA-binding protein (RBP) to regulate mRNA translation. La-related protein 1 (LARP1) has been uncovered as the mTORC1 RBP target that regulates the translation of TOP mRNAs through direct interaction [10–14]. How LARP1 impacts TOP mRNA translation remains controversial. LARP1 has been shown to stimulate TOP mRNA translation, repress their translation, and/or stabilize them [7, 10, 11, 13–20]. Even upon viral infection with DENV and SARS-CoV-2, opposing functions have been proposed for LARP1 [21, 22].

Most independent studies corroborate the model by which LARP1 acts as a repressor of TOP mRNA translation in human systems [7, 11, 13, 14, 16]. Through structural and *in vitro* biochemistry experiments, we demonstrated that the C-terminal DM15 module from humans interacts with the mRNA cap and first four nucleotides of the 5’ TOP motif to sterically occlude eIF4G, the scaffolding component of the eukaryotic translation initiation factor complex eIF4F [13]. Consistent with these data, Philippe and colleagues reported that the C-terminal half of LARP1, including the DM15 region, is necessary and sufficient for the mTORC1-dependent translation repression of TOP mRNA in human cells; further, they observed that minimal C-terminal LARP1 constructs encompassing only the DM15 region constitutively repressed TOP translation [16]. Importantly, recent evidence suggests that this cap-binding function of the DM15 region of LARP1 serves to stabilize target TOP transcripts, while 4E-BP inhibits their translation [23].

Interestingly, the components of the 5’ TOP-LARP1-TOR axis are not conserved to the same degree across Eukarya. While there are commonalities among the biological functions (i.e. GO terms) encoded by TOP mRNAs in different organisms, species-specific TOP transcripts also exist. In *Arabidopsis* for example, TOP mRNAs encode plant-specific gene products such as those involved in auxin signaling, in addition to a handful of cytosolic ribosomal proteins [24]. In instances where orthologous mRNAs are encoded as TOPs, sequence composition may vary. The evolutionary conservation of the TOP mRNA-LARP1-TOR axis is therefore not well understood. Herein, we focus on dissecting the DM15 interaction with 5’ TOP motifs using a comparative structural approach. We present co-crystal structures of *D*. *melanogaster* (*Dm* or fruit fly) and *D*. *rerio* (*Dr* or zebrafish) DM15 with a dinucleotide analog, revealing the conserved three-dimensional DM15 architecture; molecular dynamics simulations demonstrate that the N-terminus of this domain is predicted to be unstable and unfold in fruit fly. Moreover, we determine that both orthologs retain 5’ TOP motif binding activity facilitated by the same positively charged surface. We provide evidence that the TOP mRNA-LARP1-TOR regulatory axis is conserved across vertebrates and some invertebrates including zebrafish and fruit fly, respectively.

## Materials and methods

### Cloning and mutagenesis

Cloning of *H*. *sapiens* (*Hs* or human) *Hs*DM15 and *Dm*DM15 was previously described [12, 25]. *Dr*DM15 was cloned into a modified pET28a vector by Gibson Assembly (NEB) so that the resultant protein contains N-terminal His_6_-MBP tags followed by a Tobacco Etch Virus (TEV) cleavage site and Gly_6_ linker preceding the DM15 construct. A geneblock corresponding to the coding sequence of *Dr*LARP1 amino acids 754–904 (accession number F1R0U5) optimized for *E*. *coli* expression was used (Thermo Fisher Scientific). R840E/Y883A mutants were generated using site-directed mutagenesis with Pfu Turbo DNA polymerase (Agilent).

Primers for site-directed mutagenesis:

Dm-DM15_R847E_F: CGTTTTTGGAGCTTTTTTCTGGAGGAGAACTTTAACAAAAGCATG

Dm-DM15_R847E_R: CATGCTTTTGTTAAAGTTCTCCTCCAGAAAAAAGCTCCAAAAACG

Dm-DM15_Y883A_F: GGAATGTCTGTTTCGCTTTTTTAGCGCTGGCCTGGAAAAAAAGTTTC

Dm-DM15_Y883A_R: GAAACTTTTTTTCCAGGCCAGCGCTAAAAAAGCGAAACAGACATTCC

Dr-DM15_R840E_F: GCCAAGAAATGAATACCCTGTTTGAGTTTTGGAGCTTTTTTCTGCGCG

Dr-DM15_R840E_R: TCGCGCAGAAAAAAGCTCCAAAACTCAAACAGGGTATTCATTTCTTGGC

Dr-DM15_Y883A_F: AATGTCTGTTTCGCTTTTATAGCGCTGGTCTGGAACGTAAATTTCGTC

Dr-DM15_Y883A_R: GACGAAATTTACGTTCCAGACCAGCGCTATAAAAGCGAAACAGACATT

### Protein expression and purification

All DM15 constructs were expressed by autoinduction using BL21(DE3) cells as previously described [12]. Briefly, a confluent plate was scraped into 500 mL autoinduction media and grown shaking at ~180 RPM at 37°C. After 2.5–3 hours, the temperature was decreased to 18°C. Cells were incubated at 18°C and 180 rpm for 18–21 hours. Cells were harvested, flash frozen in liquid nitrogen, and stored at -80°C.

All DM15 constructs were purified similarly and as previously described with minor modifications to buffer conditions [12, 25]. All steps were performed at 4–8°C. Cells were resuspended in lysis buffer (50 mM Tris-HCl, pH 8, 400 mM NaCl, 10% glycerol, 10 mM imidazole) with a protease inhibitor cocktail including aprotinin (Gold Bio), leupeptin (RPI Research), and PMSF (Sigma). Approximately 50 mg lysozyme (Fisher) was added to facilitate lysis during resuspension. After cells were resuspended, they were lysed via sonication (Branson Sonifier 250, ~100 watts) with 2 min on, 1 min off, 50% pulse cycles in an ethanol/water ice bath. Lysate was clarified by centrifugation at 6°C for 30 min at 24,676 g to pellet cellular debris.

The cleared lysate was batch purified using Ni-NTA resin (Thermo Fisher Scientific). Ni-NTA resin was washed with 5 CV lysis buffer followed by 15 CV wash buffer (50 mM Tris-HCl, pH 8, 400 mM NaCl, 10% glycerol, 35 mM imidazole) to reduce non-specific protein binding to the resin. His_6_-MBP-DM15 and other bound proteins were eluted with 50 mM Tris-HCl, pH 8, 400 mM NaCl, 10% glycerol, 250 mM imidazole. The eluate was dialyzed with homemade TEV protease (50 mg fusion protein:4 mg TEV) for proteolytic cleavage overnight using a 10k MWCO SnakeSkin tubing (Thermo Fisher Scientific) against 50 mM Tris-HCl, pH 7 or 8, 100 mM NaCl, 10% glycerol, and 0.5 mM EDTA to reduce salt concentration and remove the His_6_-MBP tag. For *Hs*DM15 and *Dr*DM15 purifications, a pH of 8 was used. A pH of 7 was used for *Dm*DM15 buffers because of the differences in theoretical isoelectric points (ProtParam).

The next purification steps were completed using the AKTA Püre (GE) also at 4–8°C. Nucleic acid contaminants were removed by tandem ion exchange chromatography (HiTrap Q and HiTrap SP 5 mL columns, GE). Wash buffer and elution buffers were identical to dialysis buffer, with the addition of 1M NaCl to the latter. DM15 eluted from the SP column with a salt gradient between 0.1–1 M NaCl. DM15 was further purified using a hydrophobic column (5 mL HiTrap Butyl, GE). SP eluate fractions containing DM15 were brought to 1 M ammonium sulfate using concentrated ammonium sulfate and wash buffer (50 mM Tris-HCl pH 7, 1 M ammonium sulfate, 5% glycerol). DM15 was eluted from the butyl column with a gradient to 50 mM Tris-HCl, pH 7, 100 mM NaCl, and 2 mM DTT. DM15 fractions were dialyzed into crystallization buffer (10 mM or 50 mM HEPES, pH 7, 0 or 100 mM NaCl, 2 mM DTT) or storage buffer (50 mM Tris-HCl, pH 7.5, 250 mM NaCl, 25% glycerol, 2 mM DTT) overnight at 4°C. Fractions containing DM15 were concentrated to ~100–200 μM and flash frozen in liquid nitrogen for storage at -80°C or to 12.5–12.7 mg/mL for crystallization.

### Crystallization and structure determination

*Dm*DM15 and *Dr*DM15 were co-crystallized at 4.3 mg/mL and 8 mg/mL, respectively, with a dinucleotide analog, m^7^GpppC (Jena Bioscience), at a 1:1.16 molar ratio of DM15:m^7^GpppC by sitting drop vapor diffusion in a 2 μL drop. Drop ratio was 1:1 for protein-ligand complex:mother liquor. For *Dm*DM15, the reservoir solution (150 μL) contained 0.1 M Tris, pH 8.5, and 25% PEG 3350. For *Dr*DM15, the reservoir solution (150 μL) was composed of 0.1 M Tris-HCl, pH 8.5, and 20% PEG 1000. Crystals formed within a week. Crystals were cryoprotected by supplementing reservoir solution with 20% ethylene glycol (Hampton Research).

Diffraction data was collected and processed by Lilly Research Laboratories Collaborative Access Team (LRL-CAT) at the Advanced Photon Source at Argonne National Laboratory). Co-crystal structures were solved by molecular replacement with Phenix using chain A from *Hs*DM15 (PDB ID 5V87) as a search model after the removal of parts of loops [13]. Phenix and Coot were used for structural refinement and iterative building [26, 27], and Phenix was used to calculate the composite omit maps [26]. Figures were generated using PyMOL (Schrödinger, LLC. (New York, NY)). Models and data and are deposited under PDBIDs 8DIO (*D*. *rerio*) and 8DHU (*D*. *melanogaster*).

### Sequence alignments

Multiple sequence alignment was generated with Clustal Omega using sequences from Deragon [28]. The alignment was visualized using JalView [29–31].

### RNA oligonucleotide sequences

RNA oligonucleotide sequences used are as follows.

“Cap” able oligos (5’ triphosphate):

DmRPL30 42-mer:

5’ ppp-CUUUUGCCAUUGUCAGCCGACGAAGUGCUUUAACCCAAACUA 3’

ΔTOP_DmRPL30 42-mer:

5’ ppp-GAAAAGCCAUUGUCAGCCGACGAAGUGCUUUAACCCAAACUA 3’

*Hs*RPS6:

5’ ppp-CCUCUUUUCCGUGGCGCCUCGGAGGCGUUCAGCUGCUUCAAG 3’

PABPC1 42-mer:

5’ ppp-CCUUCUCCCCGGCGGUUAGUGCUGAGAGUGCGGAGUGUGUGC 3’

*Hs*RPS6_Stem:

5’ ppp-CCUCUUUUCCGCUUAUCUCUUUGAGAUAAAUGCAUAUUUUUU 3’

*Hs*PABPC1_Stem:

5’ ppp-CCUUCUCCCCGCUUAUCUCUUUGAGAUAAAUGCAUAUUUUUU

Oligos with a 5’hydroxyl:

*Hs*RPS6 5’ UTR:

5’ CCUCUUUUCCGUGGCGCCUCGGAGGCGUUCAGCUGCUUCAAG 3’

*Hs*RPS6_Stem:

5’ CCUCUUUUCCGCUUAUCUCUUUGAGAUAAAUGCAUAUUUUUU 3’

ΔTOP_*Hs*RPS6*_Stem*:

5’ GGAGAAAAGGGCUUAUCUCUUUGAGAUAAAUGCAUAUUUUUU 3’

### RNA secondary structure prediction

RNAfold was used to predict secondary structure with default parameters [32]. Both centroid and MFE predictions are shown where applicable. RNAinverse was used to design RNA oligos with a defined structure [32]. Forna was used to visualize the sequence and predicted secondary structure [33].

### RNA preparation

5’ triphosphorylated oligos were synthesized by ChemGenes. All other oligos except ΔTOP_DmRPL30 were synthesized by Sigma. ΔTOP_DmRPL30 was synthesized inhouse by *in vitro* transcription using homemade T7 RNA polymerase containing a P226L mutation as described previously [25, 34]. ΔTOP_DmRPL30 was subsequently gel purified using an 8% polyacrylamide (29:1)/7M urea/1X TBE denaturing gel, eluted passively with 10 mM sodium cacodylate, pH 6.5 (Hampton Research), and concentrated by ethanol precipitation.

### Electrophoretic mobility shift assays (EMSAs)

EMSAs were used to analyze binding assays. To visualize the RNA on a native gel, RNA oligos with a 5’ OH- were first 5’ end labelled with a 5’ phosphate using T4 polynucleotide kinase (Thermo Fisher Scientific) and [*γ*-^32^P]-ATP (Perkin Elmer). Labelled oligos were gel purified using a 10% polyacrylamide (29:1)/7M urea/1X TBE denaturing gel, eluted passively with 10 mM sodium cacodylate, pH 6.5 (Hampton Research), and concentrated by ethanol precipitation. 5’ triphosphorylated RNA oligos were capped and labelled using [α-^32^P]-GTP (Perkin Elmer) and the Vaccinia capping system (NEB). Unincorporated GTP was removed using G-25 spin columns (Cytiva).

Each binding reaction contained 125–500 total counts with final reaction conditions of 20 mM Tris-HCl, pH 8, 150 mM NaCl, 10% glycerol, 1 mM DTT, 0.5 μg tRNA (Ambion), 1 μg BSA (Invitrogen), and picomolar concentrations of RNA. To begin, RNA oligos were heated at 95°C for 1 min and snap cooled on ice for 30 min before the addition of protein. Concentrations of protein in each reaction are indicated above each EMSA lane. While the reaction incubated on ice, native 7% polyacrylamide (29:1)/0.5X TBE gels were pre-run for 20–45 min on ice with pre-chilled 0.5X TBE at 120 V. After 0.5–1 hr incubation, reactions were loaded onto the pre-run gels. Gels were run for another 52 min at 120 V and dried for 20–30 min for overnight exposure. Exposed phosphor screens were imaged using the GE Amersham Typhoon or Fujifilm FLA-5100 using default settings (4000 PMT, 100 μm resolution). ImageQuantTL (Cytiva) was used to quantify signal intensities with rolling ball subtraction to subtract background signal. GraphPad Prism was used to calculate dissociation constants by plotting fraction shifted vs protein concentration; curves were fit with the Hill slope nonlinear regression. Standard deviation of residuals was calculated as sy.x using GraphPad Prism.

### Molecular dynamics simulation and analysis

The protein atoms of chains B, A, and B of the human (PDBID 5V87:B), zebrafish (8DIO:A), and fruit fly (8DHU:B) DM15 structures, respectively, were used as starting points. The BIOVIA Discovery Studio Modelling Environment 2023 SP1 (BIOVIA) was used for system preparation and simulation [35]. Since some of these structures were missing residues at the C-terminus of the construct, and to keep the construct length consistent among organisms, the constructs simulated contained the equivalent residues of human isoform 2 [NP_056130.2] residues 796–941 (numbering as in S1A Fig, and for fruit fly DM15, the additional residue within this range was included). Any missing residues within 796–941 were either grafted from other chains within the same structure file, or inserted and then the conformation was updated using another chain within the same structure file. DM15 has been shown to be a monomer in solution [12], and so simulations were conducted for DM15 monomers. The systems (5V87:B, 8DIO:A, and 8DHU:B) were then cleaned with the Discovery Studio macromolecule Clean Protein tool [35]. The Prepare Protein protocol was then used to prepare the systems with ionic strength set to 0.15M and the forcefield set to CHARMM36 (the default pH value of 7.4 was used) [35–37]. The systems were solvated with the Solvation protocol within Discovery Studio [35]; solvent was added with the explicit periodic boundary model selected, a minimum distance from boundary set to 10, counter ions added, and the salt concentration set to 0.15M.

A series of 4 minimization steps was then performed for each system using the Discovery Studio Smart Minimizer tool [35]; max steps were set to 5000 for each minimization. Fixed atom constraints were used in the first 3 minimization steps. In the first step, only the hydrogen atoms were not constrained; in the second step, the hydrogen atoms and solvent were not constrained; in the third step, the hydrogen atoms, solvent, and protein side chains were not constrained; in the fourth minimization step, there were no constraints. A 100 ps heating step was performed for each system using the Dynamics (Heating or Cooling) protocol in Discovery Studio [35], with a target temperature of 310K [38]. This was followed by a 2 ns equilibration that was done using the Dynamics (Equilibration) protocol in Discovery Studio [35], with a target temperature of 310 K and Constant Pressure set to True [38]. For each system, a 2.1 μs NPT production simulation was then run using the Dynamics (NAMD) protocol in Discovery Studio [35] with the target temperature set to 310 K [39]. MDAnalysis 2.6.1 was used to align the trajectories by Cα and calculate the Cα RMSD with the corresponding first production frame as the reference [40]. Matplotlib [41] was used to generate the RMSD figures. All figures with simulation-derived DM15 structures were generated with VMD [42]. The CPPTRAJ program [43] within AmberTools23 [44, 45] was used to convert between simulation file types for analysis.

Difference residue-residue contact analysis and difference community analysis were performed with the difference contact analysis (dCNA) method [46, 47].

### Differential scanning fluorimetry (DSF)

Protein thermal shift assays, or differential scanning fluorimetry (DSF), was used to assess protein thermal stability in the absence and presence of ligand. Each reaction contained 5 μM DM15 ± 500 μM GTP (NEB) or m^7^GTP (P-L Biochemicals, a gift from William Merrick, Case Western Reserve University) with final reactions conditions of: 50 mM Tris-HCl, pH 8, 100 mM NaCl, 0.6 mM DTT, 10% glycerol, 10X SYPRO Orange (Thermo Fisher Scientific). Fluorescence at 570 nm was measured using the QuantStudio 3 System (Thermo Fisher Scientific) during a temperature ramp of 30–90°C. Fluorescence data were analyzed using QuantStudio Design & Analysis software (Thermo Fisher Scientific), and melting temperatures were calculated as described by the guide provided, defining the melting temperature (T_m_) as the midpoint of unfolding.

## Results

### The HEAT-like DM15 domain architecture is conserved over 780 million years of evolution

While the C-terminal DM15 region of human (*Hs*) LARP1 was predicted to be largely helical based on its sequence content, the crystal structure revealed that it is comprised of three tandem HEAT-like repeats [12], so-called because they lack the canonical motifs found in HEAT repeats. Accompanying biochemical studies demonstrated that these HEAT-like repeats bind RNA, whereas HEAT repeats are typically protein-interaction modules [48]. Interestingly, the human DM15 region shares 72% and 91% sequence identity with the fruit fly (*Dm*) and zebrafish (*Dr*) DM15 regions, respectively. We therefore hypothesized that both orthologs would retain the characteristic HEAT-like repeat architecture seen in *Hs*DM15.

**Fig 1:**
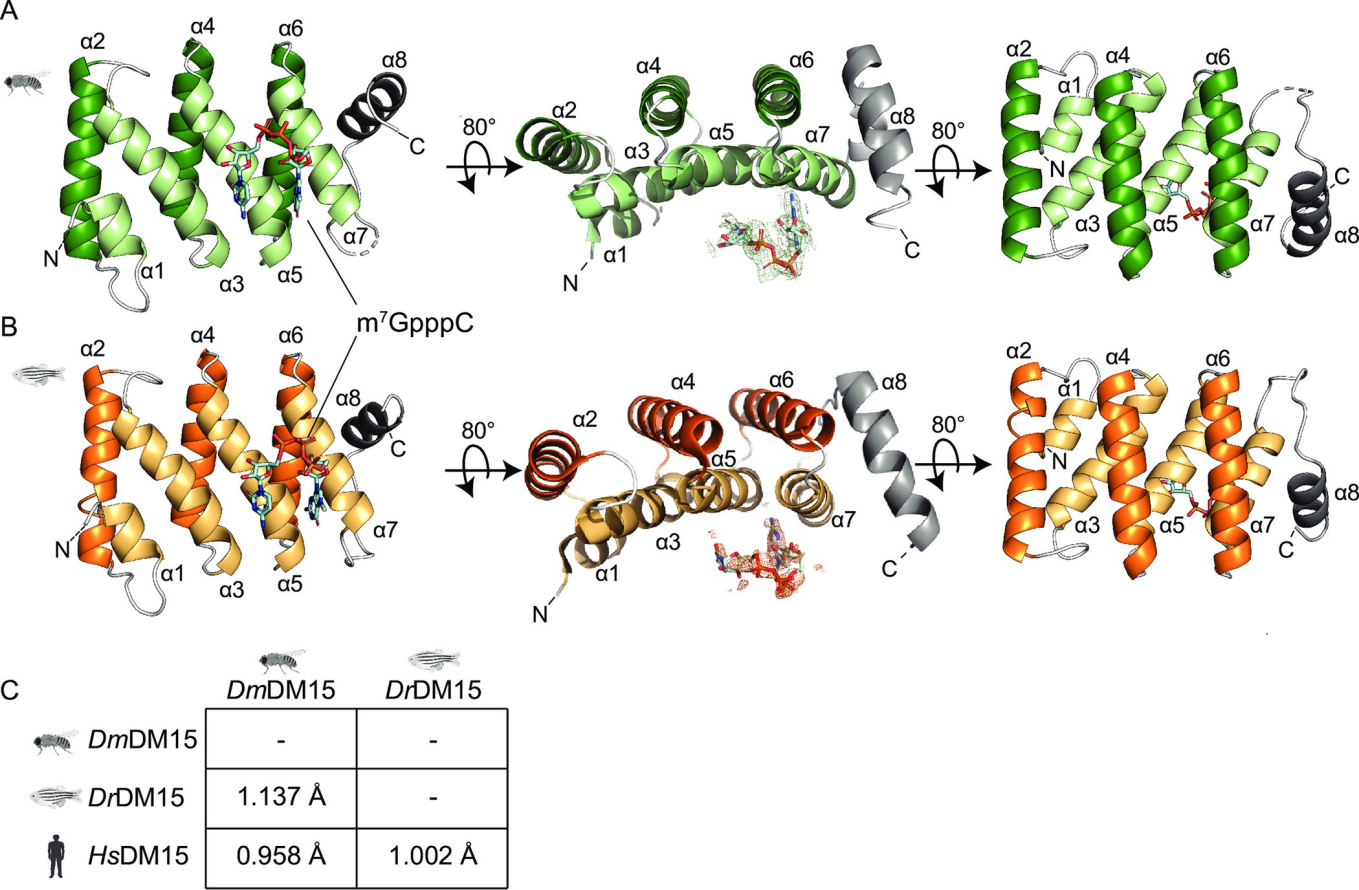
The DM15 HEAT-like fold is conserved in LARP1 from fruit fly and zebrafish. The DM15-m^7^GpppC co-crystal structures of (A) *D*. *melanogaster* (*Dm*DM15-m^7^GpppC; green) and (B) *D*. *rerio* (*Dr*DM15-m^7^GpppC; orange). The front RNA-binding helices and back helices are shown in lighter and darker colors, respectively. Composite omit maps contoured at 2σ and carved around the dinucleotide ligands are shown in the middle panes of both panels. (C) Comparison of RMSD calculated with least-squares superposition of Cα atoms using Phenix [26] among *Dm*DM15, *Dr*DM15, and *Hs*DM15 (PDBID 5V87). Organism images created with Biorender.com.

To assess whether this uniquely repurposed fold is evolutionarily conserved, we crystallized and determined the structures of both fruit fly and zebrafish DM15 regions in the presence of a dinucleotide analog, m^7^GpppC, both to 2.3 Å resolution (Table 1). We selected this ligand because the human DM15 residues that bind the m^7^GpppC dinucleotide (E886, Y883, Y922, R847, R879 in *Hs*DM15 numbering [13]) are 100% conserved among the orthologs of interest. Further, its presence stabilizes human DM15 [13], which we reasoned could aid in the crystallization of the DM15 orthologs. Indeed, both orthologs adopt a structure similar to that of human DM15 with three tandem, helix-turn-helix repeats flanked by an additional short parallel N-terminal helix and an orthogonal C-terminal helix. The repeats and additional N-terminal helix are arranged into two layers that form a concave surface that binds m^7^GpppC (Fig 1). As in the human co-crystal structure [13], m^7^GpppC is bound near the C-terminus on the positively charged swath of both orthologs where the Watson-Crick faces of both the m^7^G and the +1C are recognized through specific hydrogen bonds (Fig 1A and 1B, S2A–S2C Fig). Both structures superimpose well with human DM15 and each other with root mean square deviations (RMSD) of 0.96–1.14 Å (Fig 1C).

**Table 1:** Data-collection and refinement statistics.

PDB ID	*Dm*DM15-m^7^GpppC 8DHU	*Dr*DM15-m^7^GpppC 8DIO
*Data collection*		
Space group	P212121	P1211 or P21
Unit cell dimensions		
a, b, c (Å)	46.808 60.737 129.199 90 90 90	47.95 59.702 60.381 90 100.307 90
Unique Reflections	17090 (1695)	13814 (1391)
Resolution (Å)	44.01–2.3 (2.38–2.3)	47.18–2.3 (2.382–2.3)
R_merge_ (%)	2.0 (26.3)	1.5 (2.2)
I/σ(I)	17.0 (2.3)	28.4 (23.4)
Completeness (%)	99.7 (99.8)	91.8 (93.8)
Redundancy	2.0 (2.0)	2.0 (2.0)
CC1/2	1.000 (0.80)	0.999 (0.875)
*Refinement*		
Resolution	44.01–2.3 (2.38–2.3)	47.18–2.3 (2.38–2.3)
No. reflections	17088 (1695)	13809 (1391)
R_work_/R_free_ (%)	21.96./27/49	21.19/25.93
RMSD bond angle (°)	1.10	0.87
RMSD bond length (Å)	0.008	0.008
Average B-factor	63.34	31.85
Ramachandran favored (%)	97.3	97.6
Ramachandran outliers (%)	0	0

The majority of the residual differences among the three orthologs accumulate in the orthogonal α8 helix and helices that are not on the known RNA-binding surface, particularly in α4 and α6 (S1 Fig). One difference is apparent upon examining the hydrogen bonding network in the back layer of the DM15 helices. As compared to human DM15, fruit fly DM15 loses one net hydrogen bond between α4-α6, although the interface between α2-α4 gains a hydrogen bond (S1B Fig). Superposition of each of the three HEAT-like repeats, DM15A-C, within each structure yielded an RMSD range of 0.82–0.99 Å for fruit fly DM15, 0.69–0.77 Å for zebrafish DM15, and 0.67–0.73 Å for human DM15 (S2D–S2F Fig).

**Fig 2:**
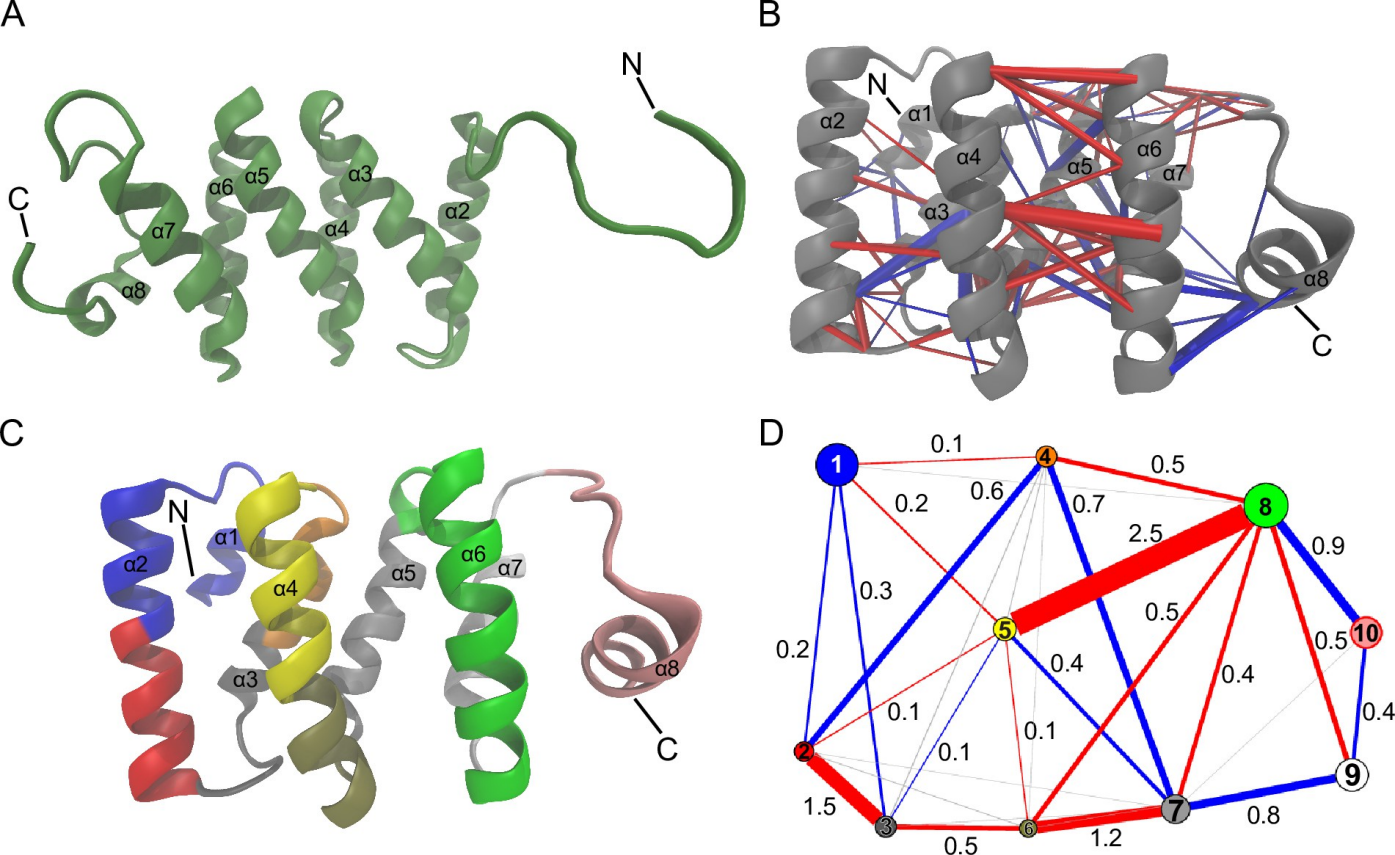
Differences in dynamics between the fruit fly, zebrafish, and human DM15 regions. (A) Simulation-derived conformation of fruit fly DM15 where α1 is unfolded. (B) Residue-residue difference contact network for the human and zebrafish DM15 simulations. Blue indicates higher probability of a residue-residue contact in the human DM15 simulation (or a lower probability in the zebrafish DM15 simulation); red, lower probability of a residue-residue contact in the human DM15 simulation (or a higher probability in the zebrafish DM15 simulation). Magnitude of the contact probability difference is indicated by the thickness of the cylinders. (C) Ten residue communities were identified from the consensus contact network, as calculated by the dCNA method [46, 47] and mapped onto the structure from a frame of the human DM15 simulation. (D) Community-community difference contact network. Each node represents the corresponding residue community in C by color. The radius of each node is proportional to the number of residues in that community. Blue lines indicate a higher probability of a community-community contact in the human DM15 simulation; red lines indicate lower probability of a community-community contact in the human DM15 simulation. Magnitude of the contact probability difference is indicated by the thickness of the lines [46, 47].

To further investigate the differences among human, fruit fly, and zebrafish DM15 domains, we conducted 2.1 μs of Molecular Dynamics Simulation for each (S3 Fig). Strikingly, the N-terminal α1 in fruit fly DM15 unfolds at ~1.5 μs (Fig 2A); the residues assigned to α1 are very dynamic after this time point. By the end of the production simulation, α1 had not refolded. There are two differences in this region in the fruit fly DM15 as compared to the vertebrate DM15 sequences: fruit fly has an alanine in position 801 (all numbering as in S1 Fig, based on human isoform 2 [NP_056130.2] for simplicity), although it is a glutamic acid in the other two organisms, and the α1-α2 loop of fruit fly DM15 has an asparagine substituted for glycine at position 807.

Zebrafish DM15 did not display such drastic dynamic deviations from the determined structure. We utilized the difference contact network analysis (dCNA) to investigate residue-residue contact and residue community contact differences between human and zebrafish DM15. Residue-residue contact differences were found within the α4- α6 interface (Fig 2B). The largest residue-residue contact difference was between 859-E899, within the α4-α6 interface; notably, residue 859 is a lysine in human DM15 and an arginine in zebrafish DM15. Another large residue-residue contact difference was between 852-D892. This is another case where in human DM15 residue 852 is a lysine, however it is an arginine in zebrafish. Additionally, the largest community-community contact difference was between residue communities 5 (yellow) and 8 (green) (Fig 2C and 2D). Unsurprisingly, these residue communities contain residues in α4 and α6, respectively. Taken together, these data demonstrate that the HEAT-like DM15 domain structure is evolutionarily conserved with predicted modest to extreme variations in protein flexibility and dynamics in the simulated conditions.

### Fruit fly and zebrafish DM15 retain 5’ TOP motif binding activities

**Fig 3:**
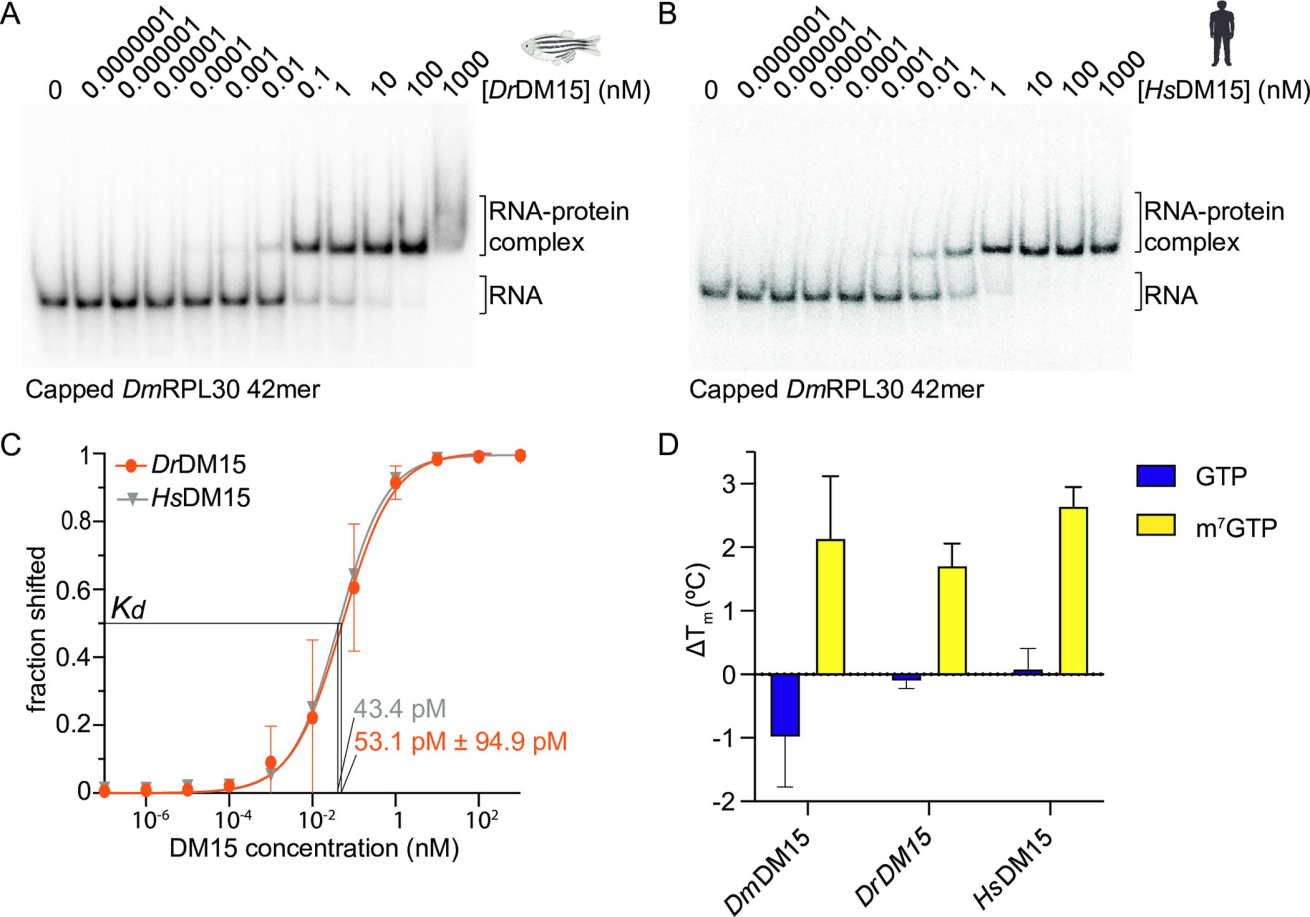
Zebrafish DM15 binds canonical 5’ TOP mRNAs. Electrophoretic mobility shift assays (EMSAs) using a canonical 5’ TOP mRNA with *Dr*DM15 (A) and *Hs*DM15 (B). (C) Quantification of EMSAs. Error bars shown for *Dr*DM15 are standard deviation of residuals reported as sy.x. Apparent binding affinities as calculated in Prism using the Hill slope nonlinear regression curve. N = 1 for *Hs*DM15; n = 6 for *Dr*Dm15 (R^2^ = 0.956). (D) Differential scanning fluorimetry of *Dm*DM15, *Dr*DM15, and *Hs*DM15 with GTP and m^7^GTP (n>3). Error bars are standard deviation. Organism images created with Biorender.com.

We previously showed that, like human DM15, fruit fly DM15 directly binds 5’ TOP mRNAs *in vitro* and *in vivo* [12, 25]. Because the direct RNA-binding residues of human DM15 are 100% conserved in zebrafish DM15, we hypothesized that zebrafish DM15 would also bind 5’ TOP mRNAs. To test this hypothesis, we used electrophoretic mobility shift assays (EMSAs) with a capped oligonucleotides containing the first 42 residues of a representative canonical TOP 5’UTR, the fruit fly RPL30 sequence. Though the sequence composition of the RPL30 5’ UTR varies across the three organisms (S4A Fig), the unifying characteristics of the pyrimidine tract are conserved, suggesting it is a good model TOP RNA with which to probe the RNA-binding profiles of the DM15 regions from the different organisms. Both zebrafish DM15 and human DM15 bound this RNA with picomolar affinity (Fig 3A–3C). To ensure we were not simply observing promiscuous binding, we eliminated the 5’TOP motif by transversion, wherein pyrimidines were changed to their purine hydrogen-bonding partner, expecting that the DM15 regions would not bind it; indeed, that is what we observed (S4B and S4C Fig). These data suggest that the zebrafish DM15 region directly binds the 5’ TOP motif. Consistent with the EMSAs and as observed in the crystal structures, thermal stabilization of all three orthologs in the presence of a cap analog (Fig 3D) further supports conserved 5’ end binding.

**Fig 4:**
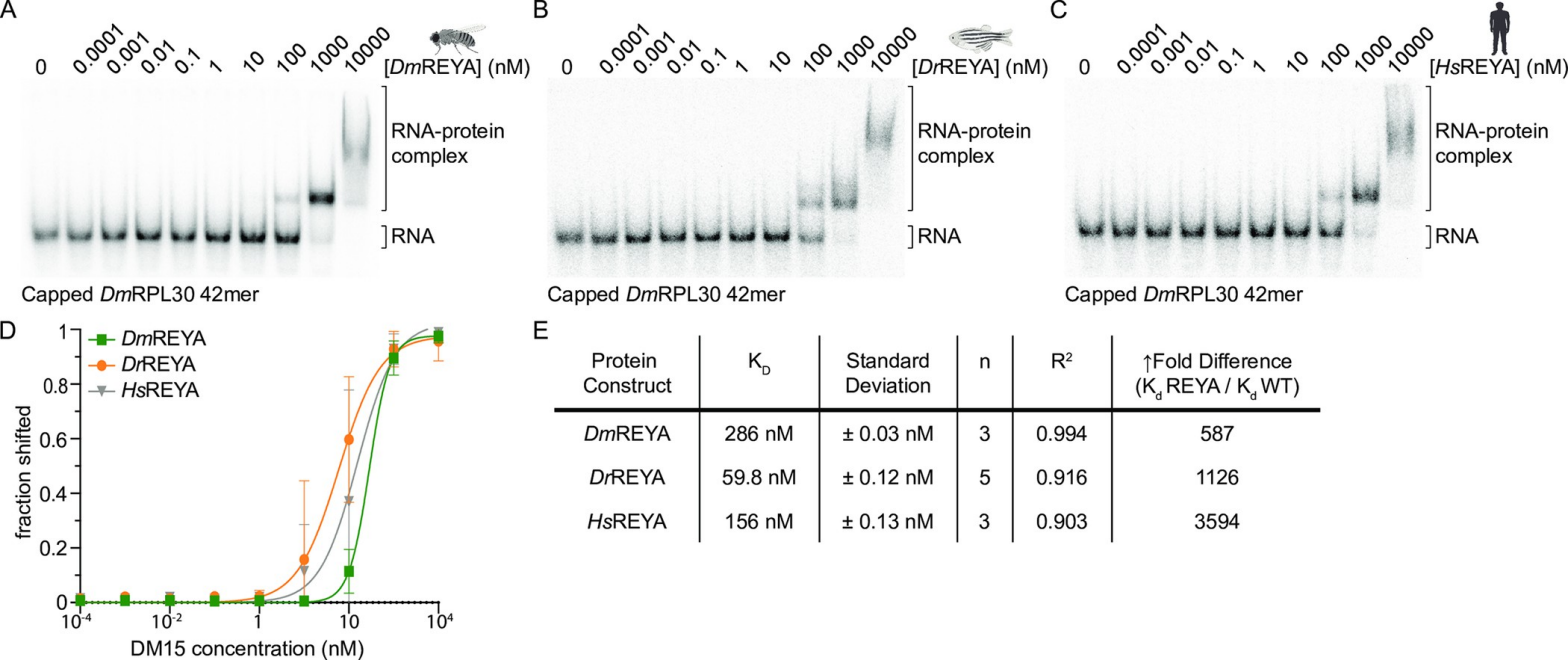
Zebrafish and fruit fly DM15 bind 5’ TOP mRNAs with their conserved positively charged surfaces. EMSAs using an oligonucleotide representing a canonical TOP mRNA with *Dm*REYA (A), *Dr*REYA (B), and *Hs*REYA (C). (D) Quantification of EMSAs. Data are fit with a nonlinear regression Hill slope curve. Error bars shown are standard deviation calculated as sy.x. (E) Apparent binding affinities and fold-change differences from WT counterpart are shown.

Given that all three orthologs bind the dinucleotide on the highly conserved, positively charged, concave surface, and they all bind 5’ TOP mRNAs, we hypothesized that fruit fly and zebrafish DM15 regions bind TOP mRNAs using the same surface as was observed for human DM15 [13]. To test this hypothesis, we mutated two key residues along the tract whose mutation in human DM15 obliterated binding to 5’ TOP mRNAs *in vitro* and in cells [13]. In the human sequence, these mutations were R840E and Y883A, and the construct was therefore called REYA [13]. All three orthologs significantly reduced binding to a capped oligonucleotide representing the *Dm*RPL30 TOP sequence with a range of 587-3594-fold difference compared to the matched wild type DM15 (Fig 4); interestingly, the REYA mutants did not completely eliminate binding as we had predicted, even for the human DM15 region. These data demonstrate that the recognition of the 5’ TOP motif is evolutionarily conserved in fruit fly and zebrafish DM15.

### The TOP motif exists in the context of a structured UTR

Having demonstrated that the LARP1 DM15 regions of evolutionarily distant organisms recognize the extreme 5’ end of TOP motifs, we sought to understand the nucleotide context of recognition. The co-crystal structure of the human DM15 region bound to RNA suggests that the DM15 region recognizes the cap and first four nucleotides of the RNA [13]. However, the observed affinities for different TOP sequences could not be predicted or rationalized based on the structures and residues that recognize these nucleotides [13]. While 5’ UTR sequences of orthologous TOP mRNAs diverge (S4 Fig for example), the nucleotides within and downstream of this tract are predicted to form similar stem-loop secondary structures (S5A and S5B Fig). Therefore, we investigated whether the sequences downstream of the polypyrimidine tract of the TOP motif influence the ability of orthologous DM15 regions to bind the motif.

**Fig 5:**
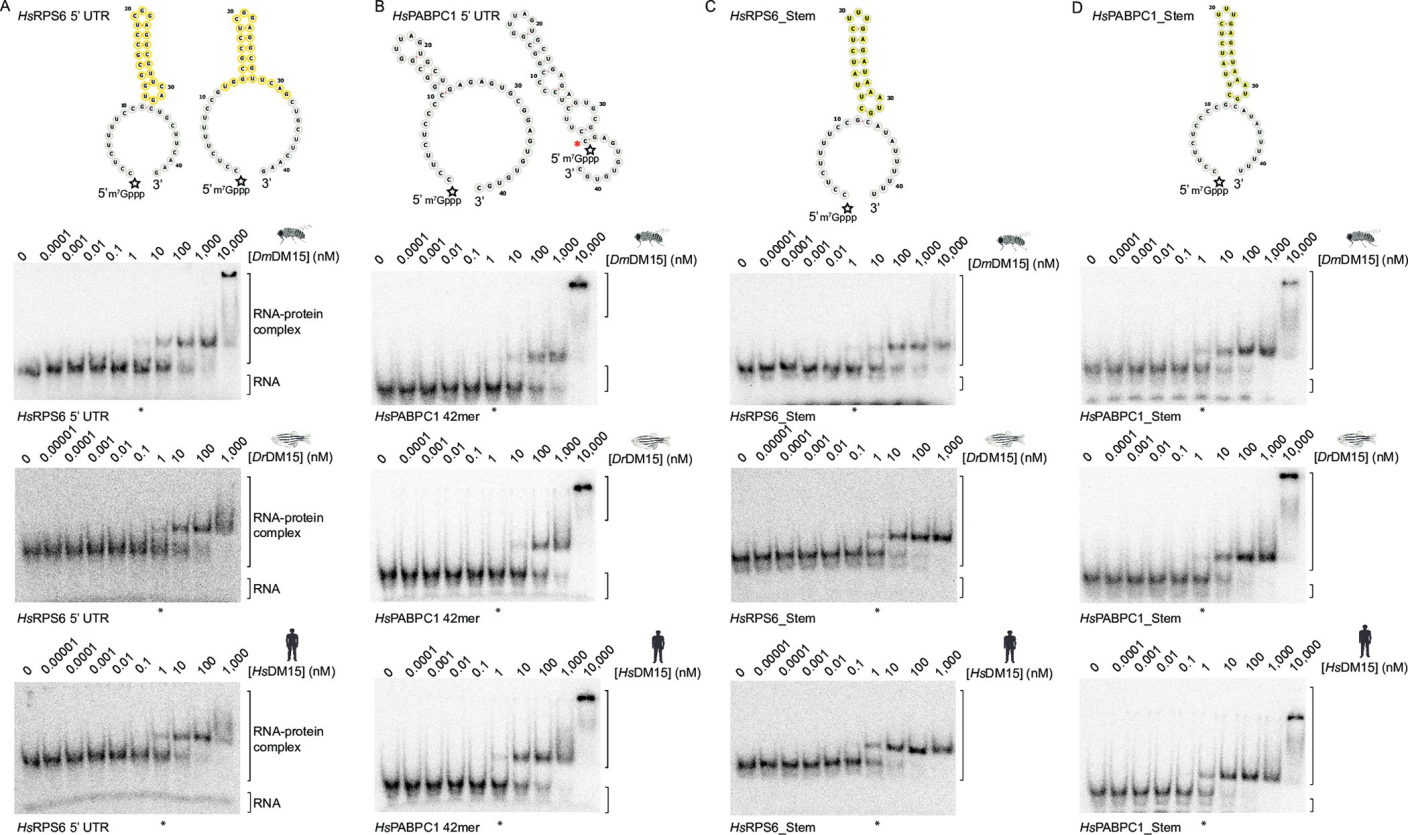
Structural features of the TOP sequence influence binding by the DM15 region from fruit fly, zebrafish, and human. **Top,** predicted RNA secondary structures of each capped RNA substrate using RNAfold, visualized by Forna [32, 33]. White stars represent the 5’7-methylguanosine cap label position. The outlined yellow nucleotides highlight the predicted stem in the natural *Hs*RPS6 5’UTR; the filled yellow nucleotides show the structure designed to mimic the natural stem in *Hs*RPS6. **Bottom,** EMSAs using A) *Hs*RPS6 42-mer, (B) *Hs*PABPC1 5’ UTR, (C) *Hs*RPS6_ Stem, and (D) *Hs*PABPC1_Stem with *Dm*DM15 (top gels), *Dr*DM15 (middle gels), and *Hs*DM15 (bottom gels) show that a structured region 3’ of the TOP motif aids in the binding interaction. Asterisks denote lanes containing 1 μM protein in each gel for ease of interpretation. The red asterisk shows the predicted fold wherein the 5’ end of *Hs*PABPC1 5’UTR is inaccessible. Organism images created with Biorender.com.

We tested capped 42-mer oligonucleotides representing the 5’UTRs of the transcripts encoding *Hs*RPS6 and *Hs*PABPC1, the latter of which was not previously a target for human DM15 [12, 13]. All three orthologs bound the 5’ UTR of *Hs*RPS6 with nanomolar apparent affinities, but did not bind as well to the *Hs*PABPC1 5’UTR 42-mer (Fig 5A and 5B and Table 2); this was even more apparent for uncapped RNA probes (S5A and S5B Fig). As before, this initially suggested that these DM15 orthologs bind some 5’TOP motifs but not all 5’TOP motifs [12, 13].

**Table 2:** Apparent binding affinities (*K*_*d*,*app*_) of the DM15 orthologs with *Hs*RPS6, *Hs*RPS6_Stem, *Hs*PABPC1, and *Hs*PABPC1_Stem RNAs.

	*K*_*d*,*app*_ ± SD (nM; n = 3)			
	*Hs*RPS6	*Hs*RPS6_Stem	*Hs*PABPC1	*Hs*PABPC1_Stem
Fruit fly	58 ± 0.02	29 ± 0.06	140 ± 0.02	6.1 ± 0.03****
Zebrafish	10 ± 0.03	7.9 ± 0.07	97 ± 0.02	6.9 ± 0.04****
Human	3.9 ± 0.2	1.9 ± 0.05	18 ± 0.06	1.5 ± 0.04****

****< 0.0001, 2-way Anova comparing each natural RNA to its engineered Stem version

Since it is generally well accepted that LARP1 regulates most, if not all, TOP mRNAs [7, 49–51], we wanted to understand this reproducible discrepancy in the *in vitro* TOP motif binding capacity: why does the DM15 region of vertebrate LARP1 interact with the TOP motif of RPS6, but not that of PABPC1? Does the predicted stem-loop structure of *Hs*RPS6 downstream of the 5’ TOP motif promote its interaction with the DM15 region (Fig 5A and S5A Fig, nucleotides circled in yellow)? Alternatively, one of the predicted structures of the *Hs*PABPC1 42-mer conceals the very 5’ end pyrimidine residues in base pairs, thereby making the single-stranded TOP motif inaccessible for recognition by the DM15 region (Fig 5B and S5B Fig, red asterisk); is the *in vitro* inability of DM15 region to bind the PABPC1 5’TOP motif simply due to issues of RNA folding?

We first asked if the structure downstream of the polypyrimidine tract is important for DM15 recognition of the RNA. To address this possibility, we designed a hairpin structure whose fold is predicted to mimic that of the *Hs*RPS6 42-mer (oligonucleotides denoted with the word “Stem;” Fig 5C and S5D Fig yellow nucleotides). When appended to the polypyrimidine tract of the *Hs*RPS6 42-mer, none of the orthologs had significantly different binding affinities as compared to the natural *Hs*RPS6 42-mer (Fig 5 and Table 2). We also tested an uncapped oligonucleotide with a transverted the 5’TOP motif, which eliminates the 5’TOP sequence, but retains the downstream structure. The orthologs did not bind as well to these oligos as they did to matched RPS6 or RPS6_Stem (S5C Fig), suggesting that affinity is not due to the direct binding of nucleotides downstream of the TOP motif.

We also tested the alternative hypothesis that the *Hs*PABPC1 polypyrimidine tract is buried within intramolecular base pairing, thereby making it inaccessible for recognition by the DM15 region. To do this, we used the designed hairpin structure (“Stem”), which is predicted to only form a hairpin at the 3’ end (Fig 5C and 5D, S5D and S5E Fig, yellow nucleotides), allowing accessibility of the polypyrimidine tract. When the 5’ TOP motif of *Hs*PABPC1 was appended to this 3’ stem structure (*Hs*PABPC1_Stem), binding by all DM15 orthologs was significantly enhanced relative to the wild type *Hs*PABPC1 RNA oligonucleotide (Fig 5 compare B with D; S5 Fig compare B with E, Table 2). In sum, these data suggest that the structural context of the 5’TOP motif is important for allowing access of the polypyrimidine tract to the DM15 region of LARP1.

## Discussion

Central to the control of ribosome biogenesis in humans, the TOP mRNA-LARP1-mTOR axis coordinates translation with environmental cues. However, all the components of this regulatory axis are not conserved to the same degree across the eukaryotic domain. TOR is ubiquitous among eukaryotic species belonging to the major clades, with the exception of obligate intracellular parasites [1]. LARP1 is similarly conserved, with representation in each of the eukaryotic supergroups [30]. Unlike for TOR and LARP1, the presence of TOP mRNAs is not as well reported; this could be due, in part, to the lack of accurate transcription start site mapping (e.g. CAGE-seq) for most organisms. It is known, however, that 1) a transcript harboring a 5’ TOP motif in one species may not harbor a 5’ TOP motif in another and 2), not all invertebrates have 5’ TOP mRNAs even where DM15-containing LARP1 and TOR both exist. Whereas this regulatory axis is conserved in plants [24], despite closer ancestral ties to humans, the TOP mRNA-LARP1-TOR axis is not conserved across invertebrates. For example, *C*. *elegans* has TOR and LARP1 orthologs, but does not have TOP mRNAs [52].

Because of LARP1’s role in the control of TOP mRNA translation in humans, we examined its orthologous biochemical functions in zebrafish (*D*. *rerio*) and fruit fly (*D*. *melanogaster*). In this study, we focused on the C-terminal DM15 region of LARP1, which is sufficient for translational repression of 5’ TOP mRNAs in both human cells and fruit flies [16, 25]. Using a comparative structural approach of the DM15 region, we show that this regulatory axis is likely conserved across vertebrates including zebrafish and some invertebrates like fruit flies, likely extending among all Coelomates.

Here we show that the fruit fly and zebrafish DM15 modules consist of HEAT-like repeats arranged in the same way as was observed for human DM15 (Fig 1 and S2 Fig). HEAT-repeats bear structural flexibility, which may facilitate plasticity in substrate binding [48, 53]. While the overall structures of all three homologs of interest remain expectedly convergent, there are indeed predicted differences in the dynamics and residue-residue contacts over time (Fig 2). As compared to the human DM15 region, zebrafish DM15 displayed greater contact probability for multiple residues in the interface between alpha helices 4 and 6 (α4-α6). The fruit fly DM15 simulation contained predicted dynamics of alpha helix (α1) that were not observed in the human and zebrafish DM15 simulations.

The N-terminus of fruit fly DM15 is predicted to be unstable, with α1 unfolding ~1.5 μs into the production simulation (Fig 2A and S3C Fig). The sequence differences at residues 801 and 807 may contribute to this instability and unfolding event. A glutamic acid in human and zebrafish DM15, residue 801 forms a salt-bridge with K804 during portions of the human and zebrafish DM15 simulations (S6A and S6B Fig); E801 in human and zebrafish DM15 also hydrogen bonds with the backbone of N796 (S6C and S6D Fig) during portions of the simulations. These interactions may contribute to the stability of α1 in human and zebrafish DM15 and are not possible in fruity fly DM15 because residue 801 is an alanine (S1A Fig). Additionally, residue 807 in the α1- α2 loop is an asparagine in fruit fly DM15, but a glycine in both human and zebrafish DM15 (S1A Fig). N807 interacted with the backbone of both A801 and E805 during portions of the simulation, which would not be possible with G807 in the human and zebrafish structure (S6E and S6F Fig). These interactions may contribute to the unfolding of fruit fly α1 by facilitating the stabilization of alternative states that culminate in the unfolding event. Additionally, the switch to a residue with more flexibility is expected to affect the energy landscape; this flexible side chain could cause instability if it were to enter the nonpolar/hydrophobic environment among α1, α2, α3, and α4.

We further demonstrated that the DM15 regions from fruit fly and zebrafish bind the 5’ cap and 5’TOP motif of TOP mRNAs using the same surface as human DM15 does (S2 Fig). These results were not particularly surprising as the deeply conserved RNA-binding residues identified in human DM15 are identical in both orthologs (S1A Fig and [28, 54]) and the human DM15-RNA co-crystal structure did not reveal interactions that would dictate preference for any particular pyrimidine sequence [13]. We also showed that the RNA-binding ability of all the DM15 region orthologs seems to be driven by affinity for the polypyrimidine rather than origin of sequence (Fig 3); human DM15, for example, binds very tightly to *Dm*RPL30.

We and others have shown that mutation of cap-binding residue Y883 and +1-nucleotide-binding residue R840 (*Hs* isoform 2 numbering) abrogates the binding of the LARP1 DM15 region to human 5’TOP motifs [12, 16, 55]. Furthermore, we previously demonstrated that the 5’TOP RNA-binding selectivity of the human LARP1 DM15 region likely originates from the cap- and +1-binding pockets [55]. That the REYA mutants of these orthologs interact relatively tightly with the *Dm*RPL30 TOP sequences (Fig 4) was therefore very surprising. While the mutants had far weaker interaction (~600–3,500-fold) with this RNA sequence than the wild-type DM15 regions did, they still bound with relatively high affinities. Perhaps the ability of the REYA mutants to interact strongly and specifically with TOP sequences suggests that other residues in the RNA-binding cleft should be assessed for their contributions to binding selectivity.

Because the DM15 interaction with the 5’ TOP motif did not appear to discriminate between cytosine and uracil beyond the +1 position by analyses of the orthologous co-crystal structures, we interrogated the secondary structures of 5’ TOP mRNAs, with a focus on the nucleotides 3’ to the initial pyrimidine tract. We showed that a structured region 3’ of the TOP motif can be important for interaction with the DM15 regions from all three orthologs (Fig 5 and S5 Fig). We suspect that the secondary structure facilitates the accessibility of the TOP motif, enabling DM15 recognition. Indeed, recent work examining the role of LARP1 in TOP mRNA translation dynamics identified PABPC1 mRNA as a transcript that was unaffected by loss of LARP1 [23], suggesting that the putative RNA structure affecting the *in vitro* interaction with the DM15 region might extend into a more biologically relevant context.

Altogether, we have demonstrated that the first half of the TOP mRNA-LARP1-TOR regulatory axis identified in humans is conserved evolutionarily in fruit fly and zebrafish. While we did not experimentally explore whether TOR phosphorylates LARP1 to control 5’ TOP translation in fruit fly and zebrafish, over 50% of the serine/threonine rapamycin-sensitive phosphorylation sites identified in mouse is conserved in both [56]. Future work should be done to address the second arm of this regulatory axis to yield a more complete picture of its evolution. Indeed, further investigation into the evolution of the LARP superfamily of proteins with their RNA targets and protein-binding partners holds great potential for expanding our understanding of post-transcriptional control of gene expression, noncoding RNA processing, and mRNA fate.

## Supporting information

S1 Fig The residues of DM15 are 72% identical among fruit fly, zebrafish, and human. (A) Sequence alignment of the LARP1 DM15 region from the three organisms addressed in this paper with alpha helices denoted. Depth of color underneath each residue indicates conservation. Numbering based on the human LARP1 isoform2 sequence (NP_056130.2). (B) Small changes underly the inter-repeat hydrogen bonding network between *Hs*DM15 (grey) and *Dm*DM15 (green). Left, there are fewer hydrogen bonds (dotted lines) between α2-α4 in *Dm*DM15 than in *Hs*DM15. Right, there are more salt bridges (dotted lines) between α4-α6 in *Dm*DM15 than in *Hs*DM15.
(TIF)

S2 Fig The electrostatic surface potential of the three orthologs is similar. (A) *Dm*DM15, (B) *Dr*DM15, and (C) *Hs*DM15 (PDBID 5V7C). All were superimposed on the *Hs*DM15-RNA co-crystal structure (PDBID 5V7C). The ligands from the alignments are shown in sticks; the blue and red surfaces represent positive and negative electrostatic surface potential, respectively, as calculated by PyMOL. (D-F) Superposition of HEAT-like DM15 repeats A, B, and C from (D) *Dm*DM15, (E) *Dr*DM15, (F) *Hs*DM15.
(TIF)

S3 Fig Molecular dynamics simulation RMSD analyses. (A-C) The Cα RMSD analysis of the (A) human, (B) zebrafish, and (C) fruit fly LARP1 DM15 simulation data. The Cα RMSD with the corresponding first production frame as the reference, raw values in blue and the running average in yellow (window of 5000).
(TIF)

S4 Fig Zebrafish DM15 binds the 5’ cap and TOP motif. (A) Sequence of RPL30 5’ UTR from each organism colored by nucleotide identity. EMSAs using a 5’ TOP mRNA substrate containing a transverted TOP motif with *Dr*DM15 (B) and *Hs*DM15 (C).
(TIF)

S5 Fig DM15 orthologs bind the pyrimidine tract of *Hs*RPS6. (A-C) Predicted RNA secondary structures of each uncapped RNA substrate using RNAfold, visualized by Forna, and colored as in Fig 5 [32, 33]. Bottom, EMSAs using (A) *Hs*RPS6 42-mer, (B) *Hs*PABPC1 5’ UTR, (C) ΔTOP_*Hs*RPS6_Stem, (D) *Hs*RPS6_ Stem, and (E) *Hs*PABPC1_Stem uncapped RNA substrates with *Dm*DM15 (top panel), *Dr*DM15 (middle panel), and *Hs*DM15 (bottom panel). Asterisks denote lanes containing 1 μM protein in each gel for ease of interpretation. Organism images created with Biorender.com.
(TIF)

S6 Fig Interactions within LARP1 DM15 α1 and the α1- α2 loop. (A, B) Salt bridge between E801 and K804 observed during the molecular dynamics simulations of (A) human and (B) zebrafish LARP1 DM15. (C, D) Hydrogen bond observed between E801 and the backbone of N796 during the molecular dynamics simulations of (C) human and (D) zebrafish LARP1 DM15. These interactions would not be possible in fruit fly LARP1 DM15, due to there being an alanine at position 801. (E, F) The hydrogen bonds observed between N807 and the backbones of (E) A801 and (F) E805 during the molecular dynamics simulation of fruity fly LARP1 DM15. These interactions would not be possible in human and zebrafish LARP1 DM15 due to there being a glycine at position 807 in these two organisms.
(TIF)

S1 Raw data Raw image files. The uncropped scans for gels shown in Figs 3–5 and S4, S5 Figs are compiled herein. Gels are labeled with appropriate figure and panel, protein, and RNA.
(PDF)
