# Supplementary figures and images for: Comparative analysis of the LARP1 C-terminal DM15 region through Coelomate evolution
Source: PLoS One. 2024 Aug 27;19(8):e0308574. doi: 10.1371/journal.pone.0308574 (PMC11349179; doi:10.1371/journal.pone.0308574)

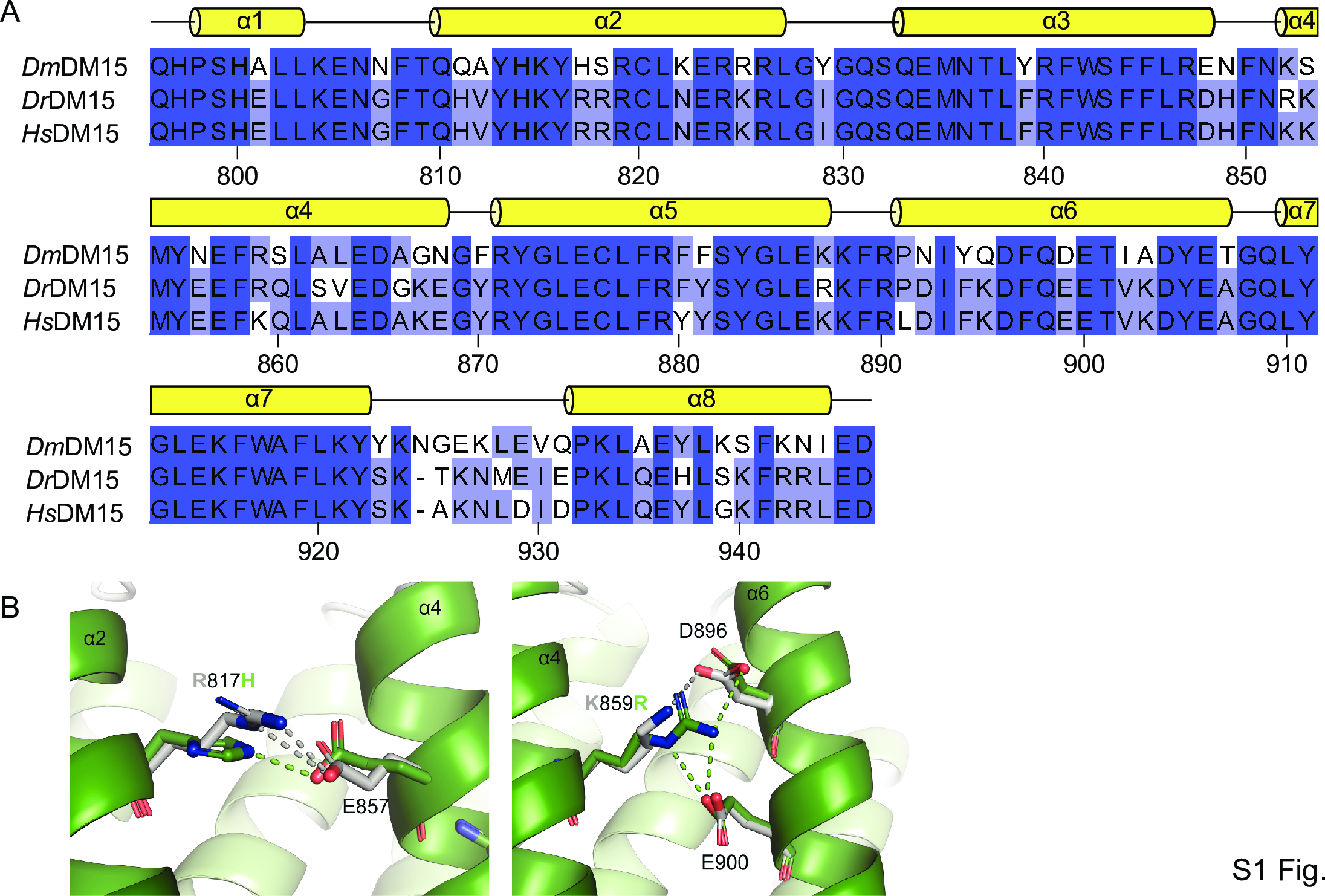

Supplement: S1 Fig — (A) Sequence alignment of the LARP1 DM15 region from the three organisms addressed in this paper with alpha helices denoted. Depth of color underneath each residue indicates conservation. Numbering based on the human LARP1 isoform2 sequence (NP_056130.2). (B) Small changes underly the inter-repeat hydrogen bonding network between HsDM15 (grey) and DmDM15 (green). Left, there are fewer hydrogen bonds (dotted lines) between α2-α4 in DmDM15 than in HsDM15. Right, there are more salt bridges (dotted lines) between α4-α6 in DmDM15 than in HsDM15. (TIF) [file pone.0308574.s001.tif]

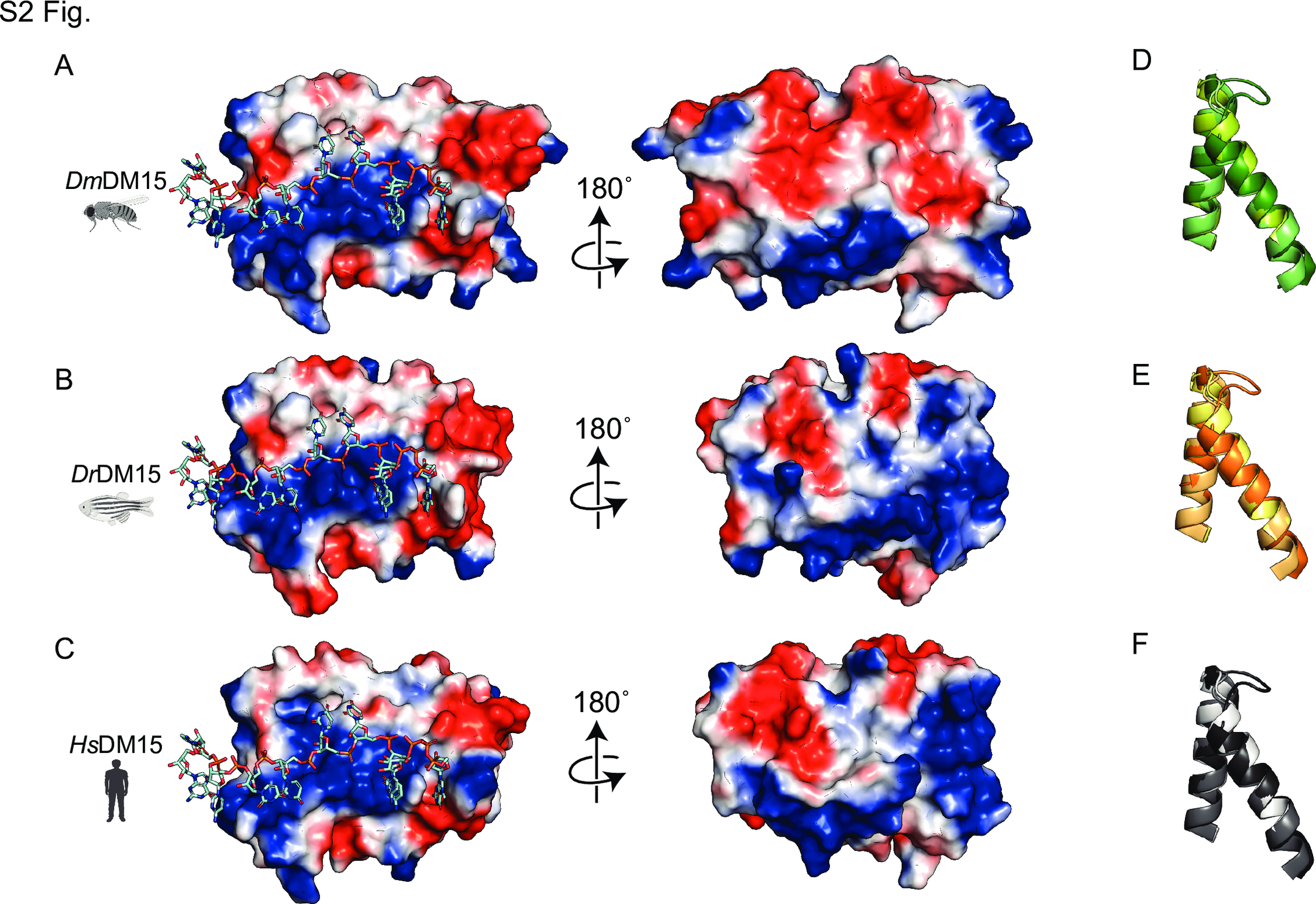

Supplement: S2 Fig — (A) DmDM15, (B) DrDM15, and (C) HsDM15 (PDBID 5V7C). All were superimposed on the HsDM15-RNA co-crystal structure (PDBID 5V7C). The ligands from the alignments are shown in sticks; the blue and red surfaces represent positive and negative electrostatic surface potential, respectively, as calculated by PyMOL. (D-F) Superposition of HEAT-like DM15 repeats A, B, and C from (D) DmDM15, (E) DrDM15, (F) HsDM15. (TIF) [file pone.0308574.s002.tif]

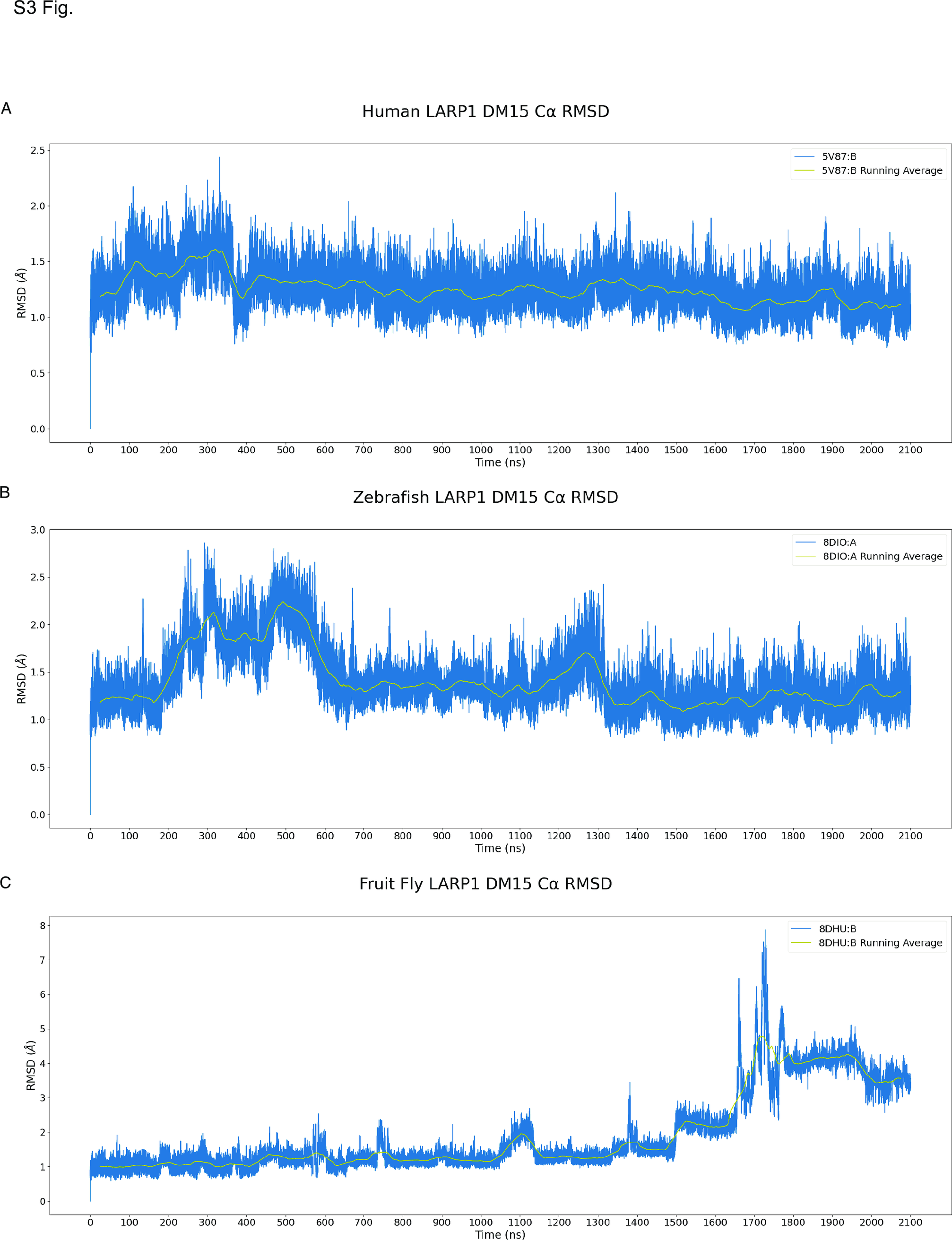

Supplement: S3 Fig — (A-C) The Cα RMSD analysis of the (A) human, (B) zebrafish, and (C) fruit fly LARP1 DM15 simulation data. The Cα RMSD with the corresponding first production frame as the reference, raw values in blue and the running average in yellow (window of 5000). (TIF) [file pone.0308574.s003.tif]

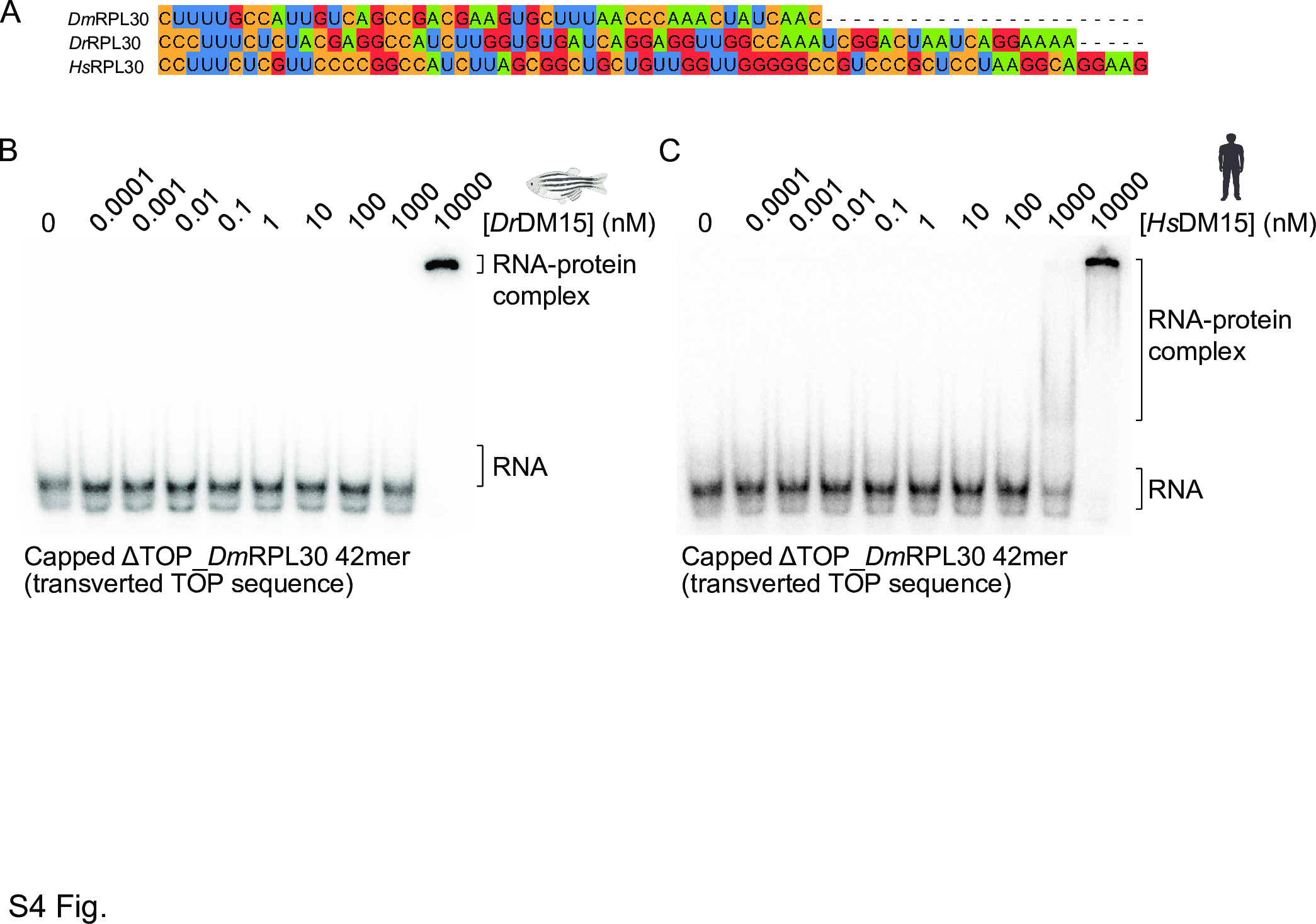

Supplement: S4 Fig — (A) Sequence of RPL30 5’ UTR from each organism colored by nucleotide identity. EMSAs using a 5’ TOP mRNA substrate containing a transverted TOP motif with DrDM15 (B) and HsDM15 (C). (TIF) [file pone.0308574.s004.tif]

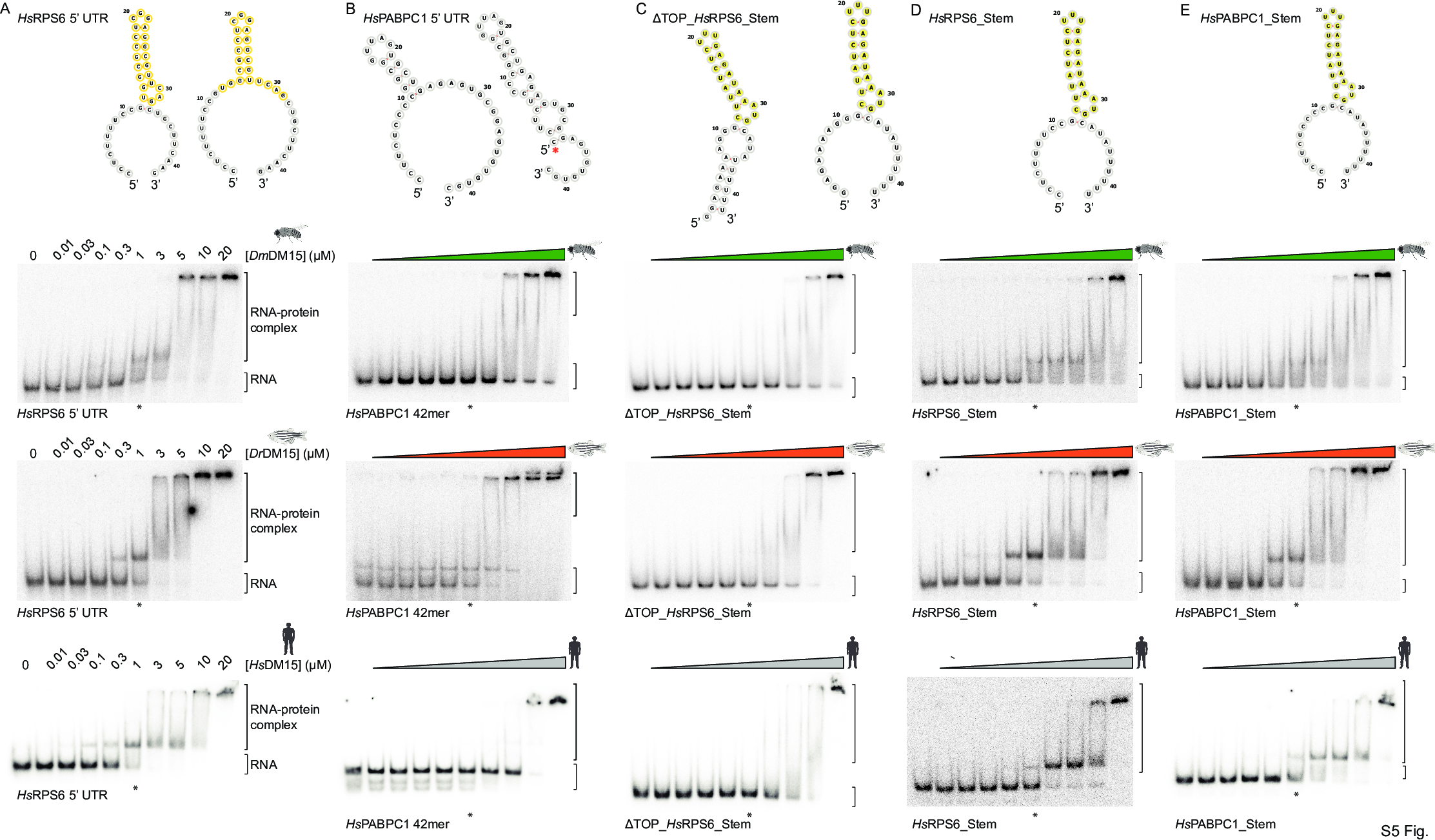

Supplement: S5 Fig — (A-C) Predicted RNA secondary structures of each uncapped RNA substrate using RNAfold, visualized by Forna, and colored as in Fig 5 [32, 33]. Bottom, EMSAs using (A) HsRPS6 42-mer, (B) HsPABPC1 5’ UTR, (C) ΔTOP_HsRPS6_Stem, (D) HsRPS6_ Stem, and (E) HsPABPC1_Stem uncapped RNA substrates with DmDM15 (top panel), DrDM15 (middle panel), and HsDM15 (bottom panel). Asterisks denote lanes containing 1 μM protein in each gel for ease of interpretation. Organism images created with Biorender.com. (TIF) [file pone.0308574.s005.tif]

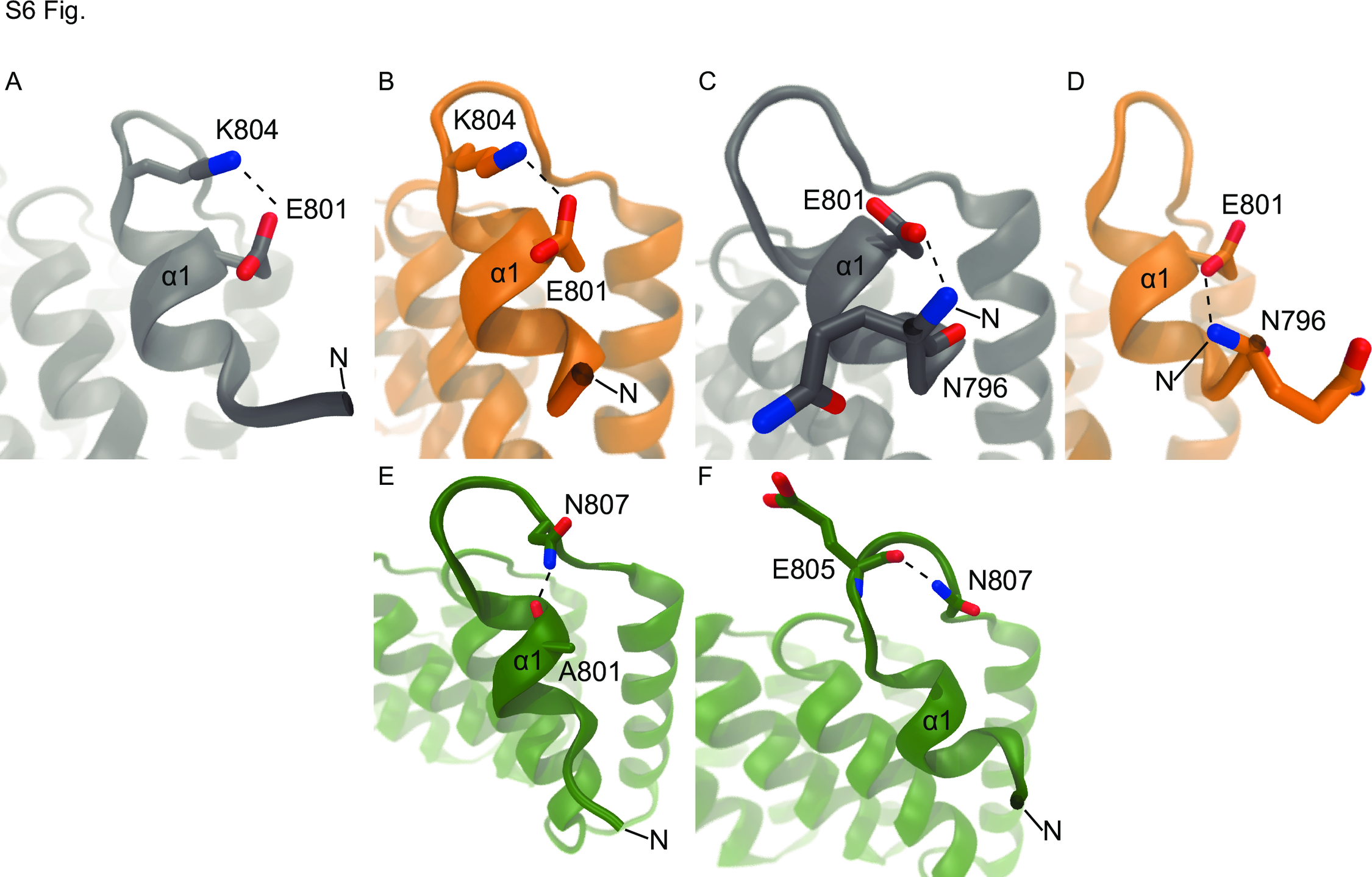

Supplement: S6 Fig — (A, B) Salt bridge between E801 and K804 observed during the molecular dynamics simulations of (A) human and (B) zebrafish LARP1 DM15. (C, D) Hydrogen bond observed between E801 and the backbone of N796 during the molecular dynamics simulations of (C) human and (D) zebrafish LARP1 DM15. These interactions would not be possible in fruit fly LARP1 DM15, due to there being an alanine at position 801. (E, F) The hydrogen bonds observed between N807 and the backbones of (E) A801 and (F) E805 during the molecular dynamics simulation of fruity fly LARP1 DM15. These interactions would not be possible in human and zebrafish LARP1 DM15 due to there being a glycine at position 807 in these two organisms. (TIF) [file pone.0308574.s006.tif]
