# Supplementary material for: Comparative analysis of the LARP1 C-terminal DM15 region through Coelomate evolution
Source: PLoS One. 2024 Aug 27;19(8):e0308574. doi: 10.1371/journal.pone.0308574 (PMC11349179; doi:10.1371/journal.pone.0308574)
Supplement: S1 Raw data — The uncropped scans for gels shown in Figs 3–5 and S4, S5 Figs are compiled herein. Gels are labeled with appropriate figure and panel, protein, and RNA. (PDF) [file pone.0308574.s007.pdf]

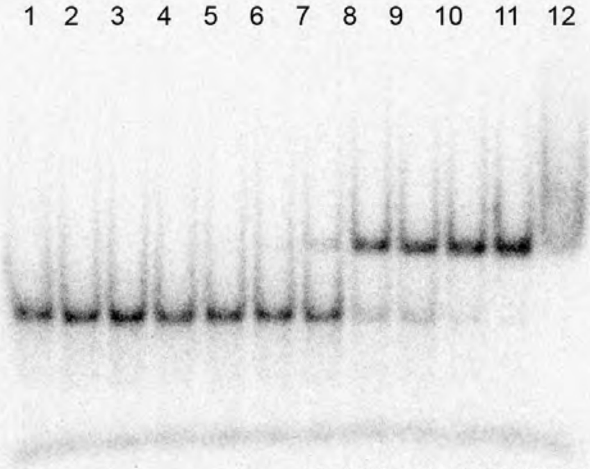

RNA: capped
DmRPL30
42-mer

Dr (Zebrafish) DM15 (nM)

1. 0	5. 1 x 10^-3	10. 10
2. 1 x 10^-7	6. 1 x 10^-2	11. 100
3. 1 x 10^-6	7. 1 x 10^-1	12. 1000
4. 1 x 10^-5	8. 1	

Figure 3A

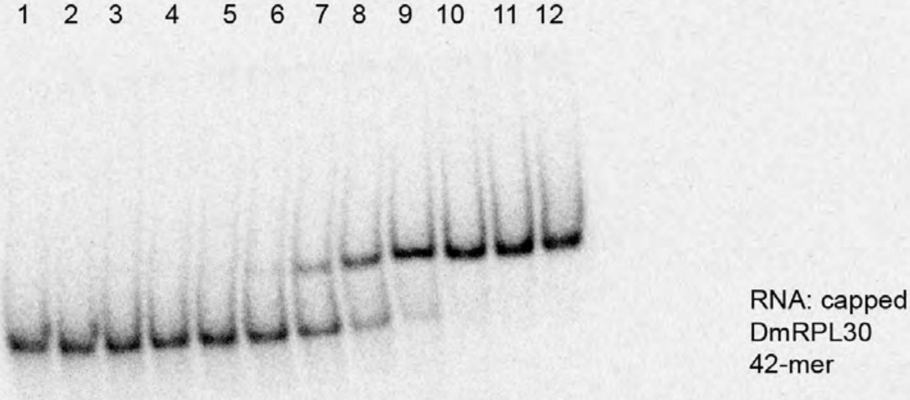

HsDM15 (nM)

1. 0
2. 1 x 10^-7
3. 1 x 10^-6
4. 1 x 10^-5
5. 1 x 10^-3
6. 1 x 10^-2
7. 1 x 10^-1
8. 1
10. 10
11. 100
12. 1000

Figure 3B

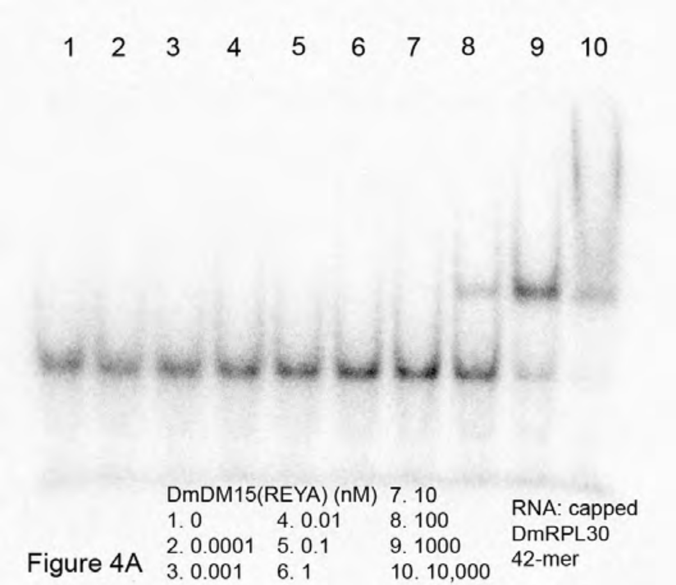
1 2 3 4 5 6 7 8 9 10
DmDM15(REYA) (nM)
1. 0
2. 0.0001
3. 0.001
4. 0.01
5. 0.1
6. 1
7. 10
8. 100
9. 1000
10. 10,000
RNA: capped DmRPL30 42-mer

Figure 4A

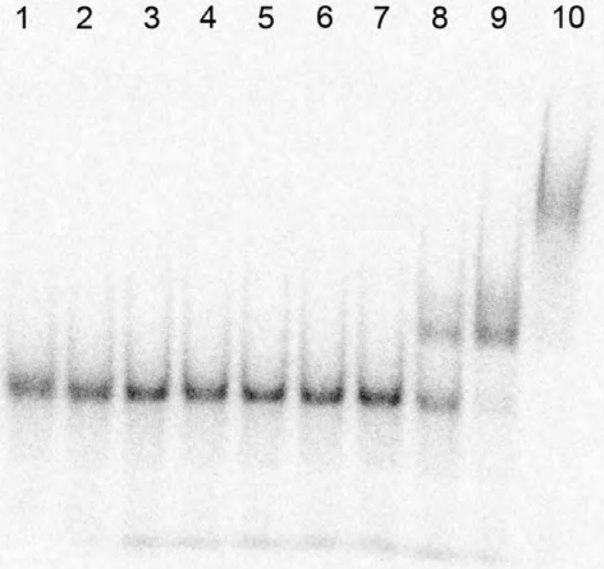

Dr (zebrafish)DM15(REYA) (nM)
1. 0
2. 0.0001
3. 0.001
4. 0.01
5. 0.1
6. 1
7. 10
8. 100
9. 1000
10. 10,000

RNA: capped
DmRPL30
42-mer

Figure 4B

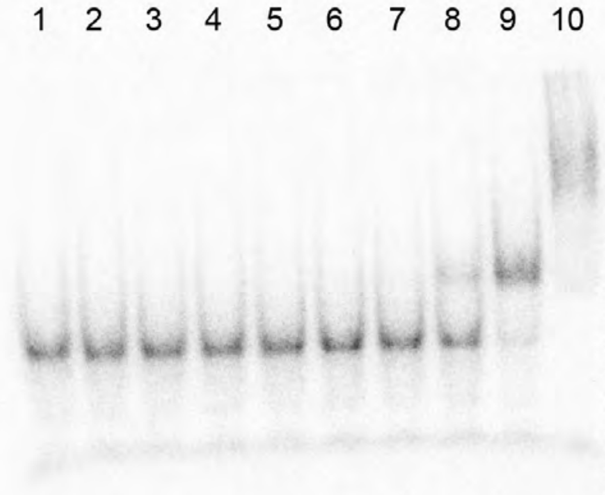

HsDM15(REYA) (nM)
1. 0
2. 0.0001
3. 0.001
4. 0.01
5. 0.1
6. 1
7. 10
8. 100
9. 1000
10. 10,000

RNA: capped
DmRPL30
42-mer

Figure 4C

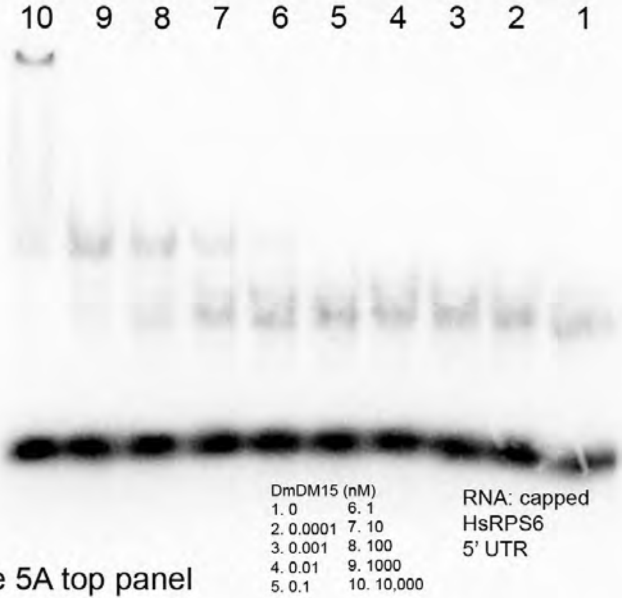

Figure 5A top panel

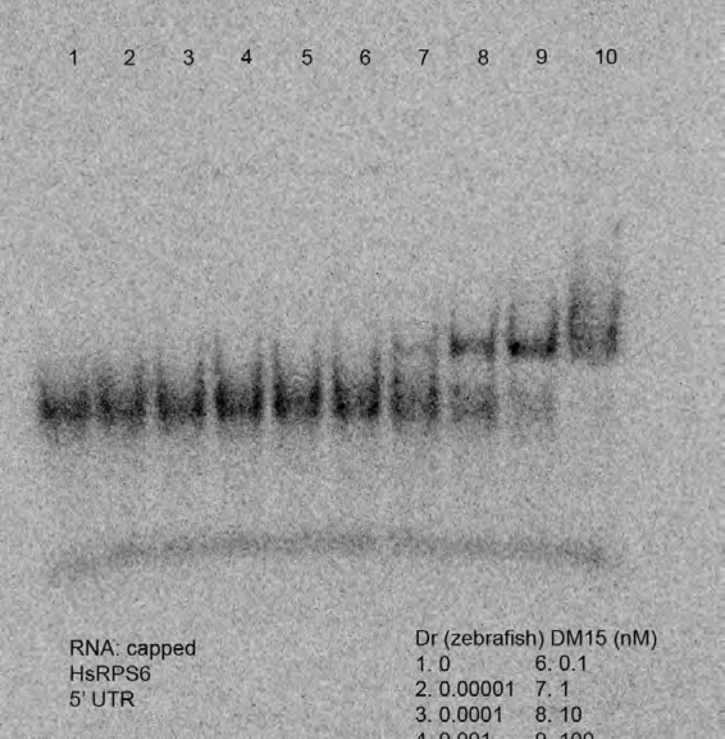

Figure 5A middle panel

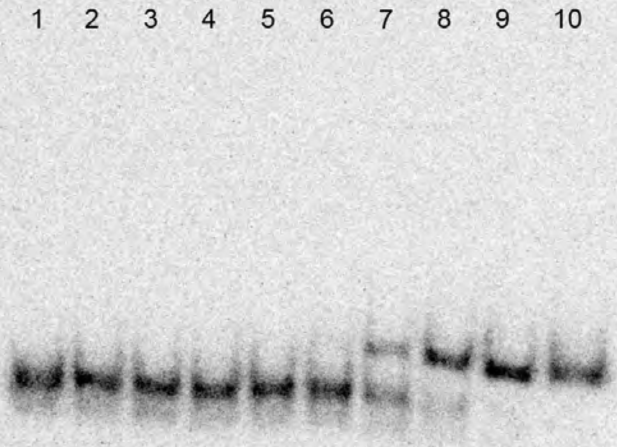

RNA: capped
HsRPS6
5' UTR

Figure 5A bottom panel

HsDM15 (nM)

1. 0	6. 0.1
2. 0.00001	7. 1
3. 0.0001	8. 10
4. 0.001	9. 100
5. 0.01	10. 1,000

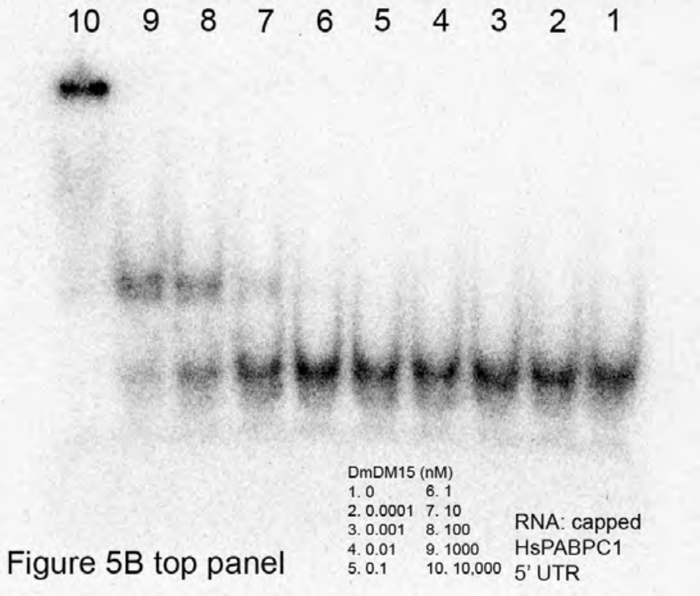

Figure 5B top panel

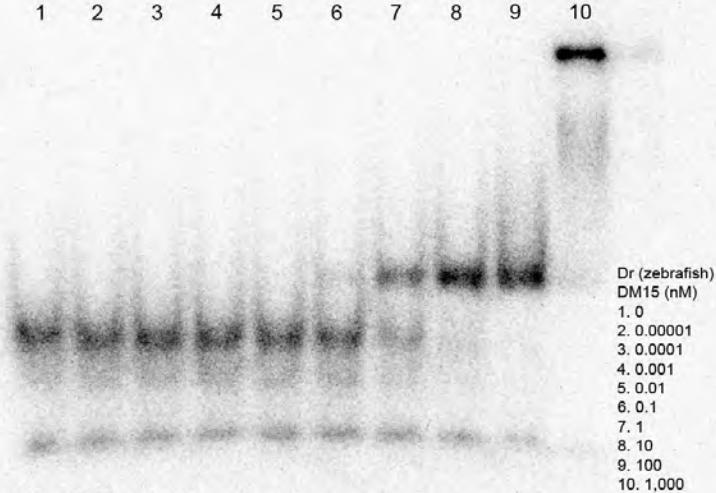

Figure 5B middle panel

RNA: capped HsPABPC1 5' UTR

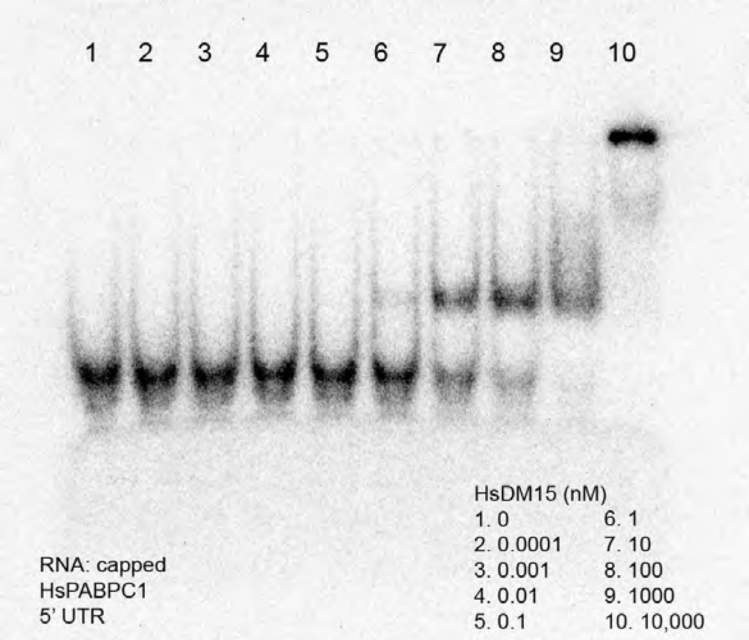

Figure 5B bottom panel

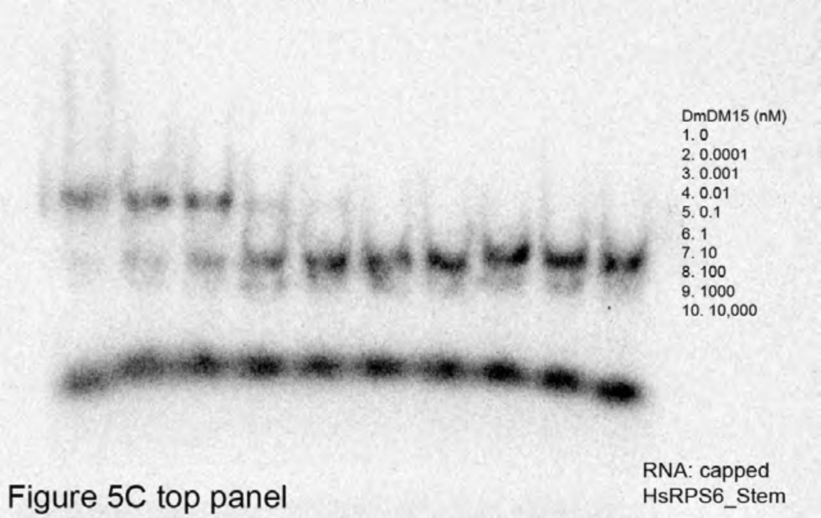

Figure 5C top panel

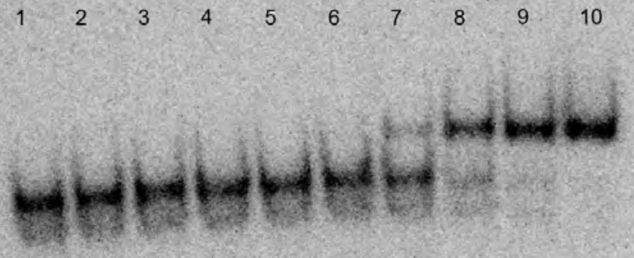

RNA: capped HsRPS6_Stem

Dr (zebrafish)
DM15 (nM)

1. 0
2. 0.00001
3. 0.0001
4. 0.001
5. 0.01
6. 0.1
7. 1
8. 10
9. 100
10. 1,000

Figure 5C middle panel

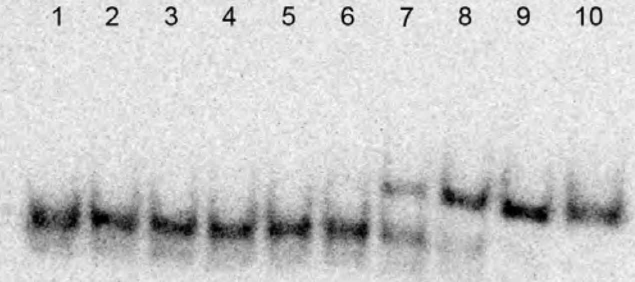

RNA: capped
HsRPS6_Stem

HsDM15 (nM)

1. 0	6. 0.1
2. 0.00001	7. 1
3. 0.0001	8. 10
4. 0.001	9. 100
5. 0.01	10. 1,000

Figure 5C bottom panel

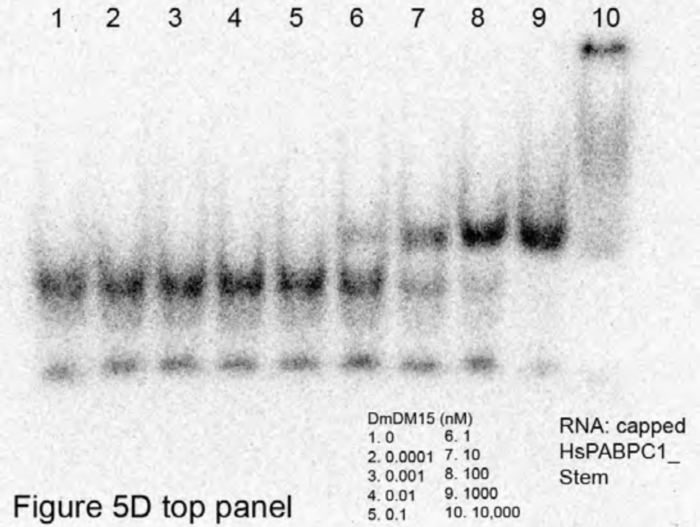

Figure 5D top panel

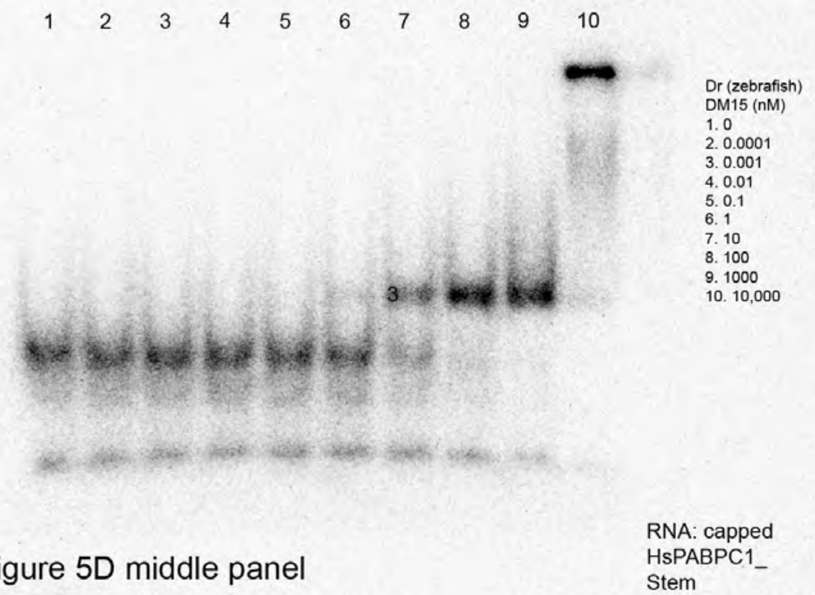

Figure 5D middle panel

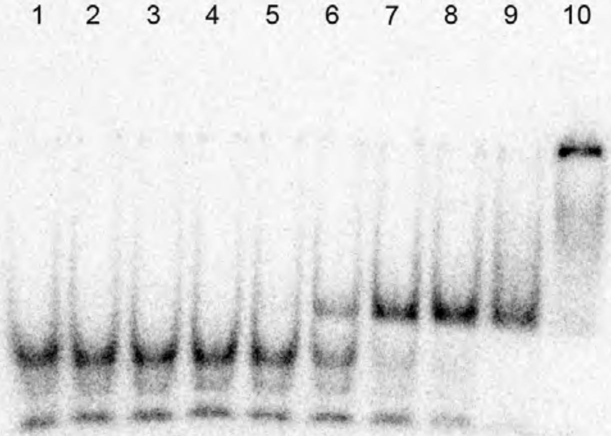

RNA: capped
HsPABPC1_Stem

HsDM15 (nM)
1. 0
2. 0.0001
3. 0.001
4. 0.01
5. 0.1
6. 1
7. 10
8. 100
9. 1,000
10. 10,000

Figure 5D bottom panel

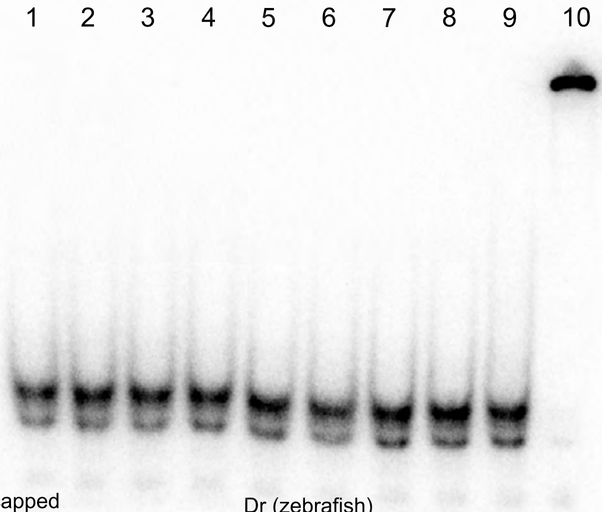

RNA: capped
deltaTOP_DmRPL30 42-mer

Dr (zebrafish)
DM15 (nM)
1. 0
2. 0.0001
3. 0.001
4. 0.01
5. 0.1
6. 1
7. 10
8. 100
9. 1,000
10. 10,000

S4 Fig.B

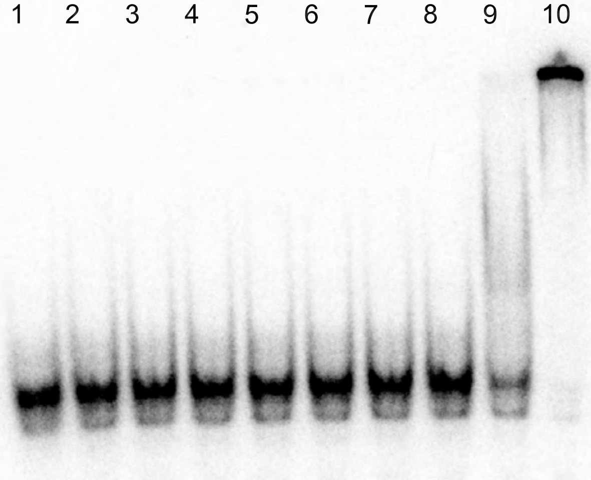

RNA: capped
deltaTOP_DmRPL30 42-mer

HsDM15 (nM)
1. 0
2. 0.0001
3. 0.001
4. 0.01
5. 0.1
6. 1
7. 10
8. 100
9. 1,000
10. 10,000

S4 Fig.C

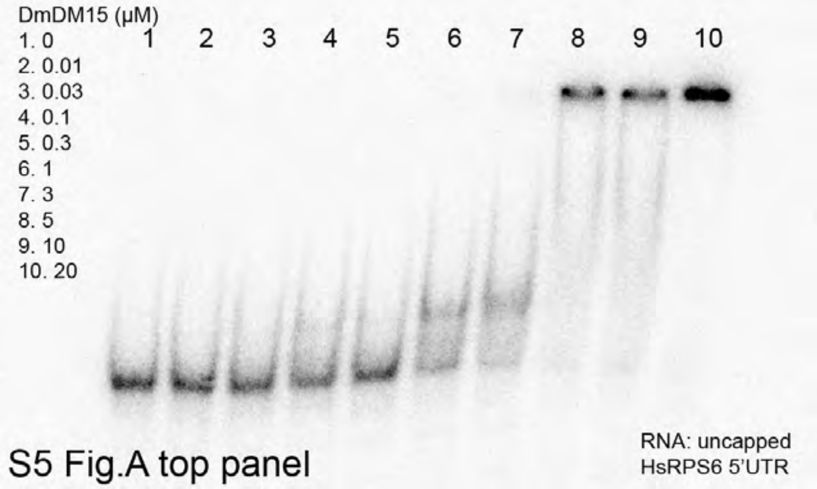

S5 Fig.A top panel

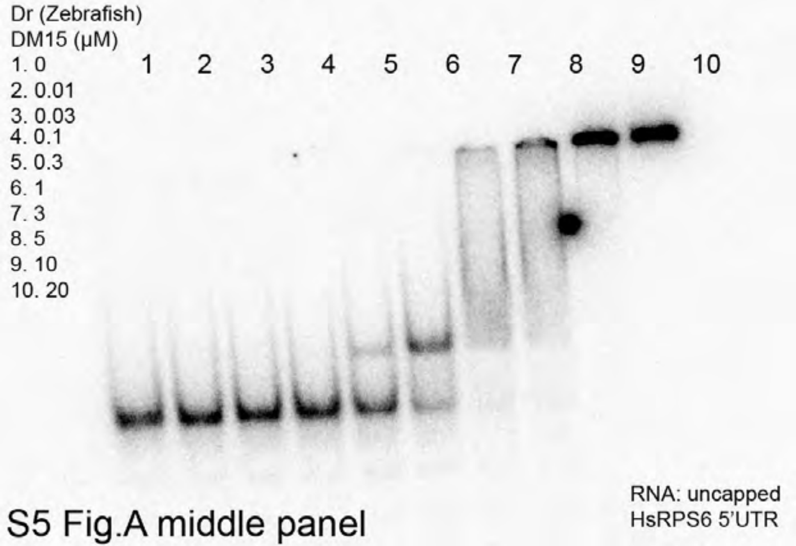

S5 Fig.A middle panel

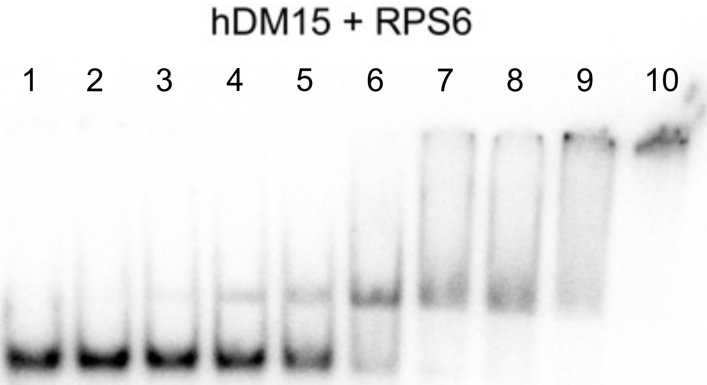

RNA: uncapped
HsRPS6 5'UTR

HsDM15 (μM)
1. 0
2. 0.01
3. 0.03
4. 0.1
5. 0.3
6. 1
7. 3
8. 5
9. 10
10. 20

Fig S5A bottom panel

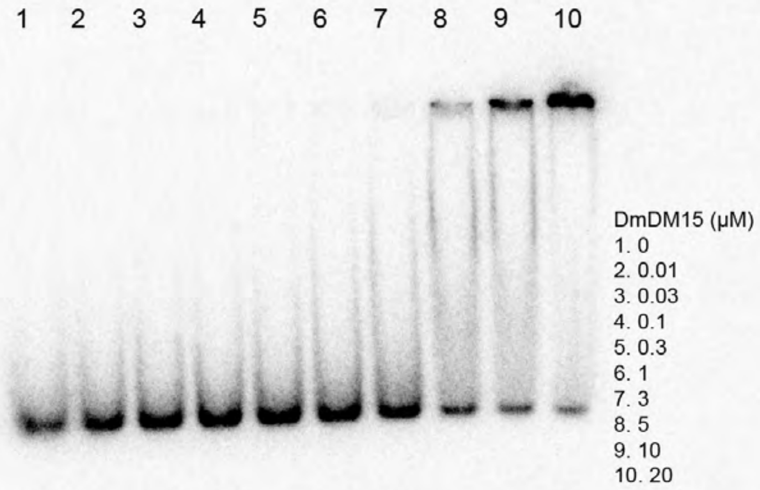

S5 Fig.B top panel

RNA: uncapped
HsPABPC1 42-mer

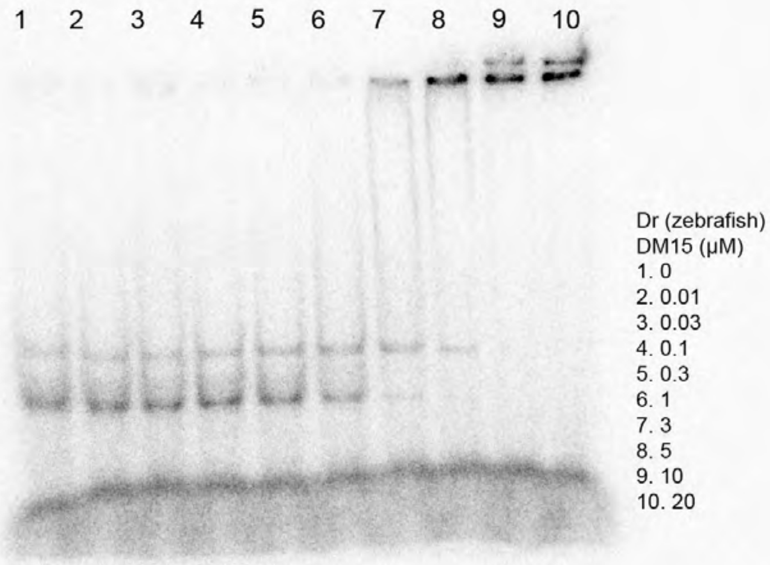

S5 Fig.B middle panel

RNA: uncapped
HsPABPC1 42-mer

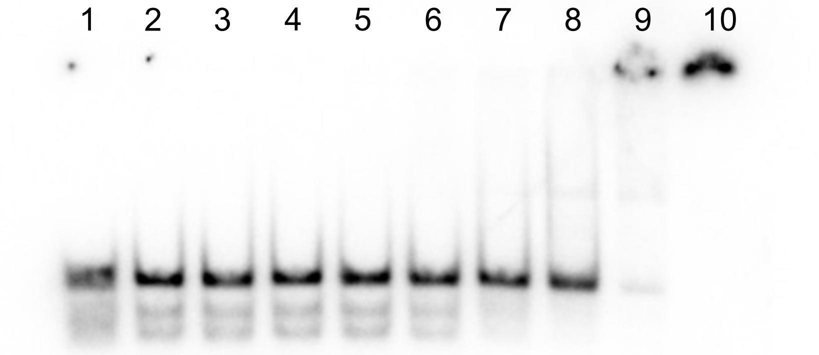

RNA: uncapped
HsPABPC1 42-mer
Fig S5B bottom panel

HsDM15 (μM)
1. 0
2. 0.01
3. 0.03
4. 0.1
5. 0.3
6. 1
7. 3
8. 5
9. 10
10. 20

DmDM15 (μM)
1. 0
2. 0.01
3. 0.03
4. 0.1
5. 0.3
6. 1
7. 3
8. 5
9. 10
10. 20

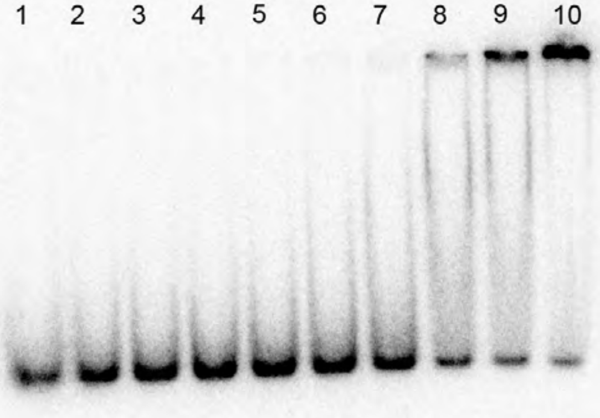

S5 Fig.C top panel

RNA: uncapped deltaTOP-HsRPS6-Stem

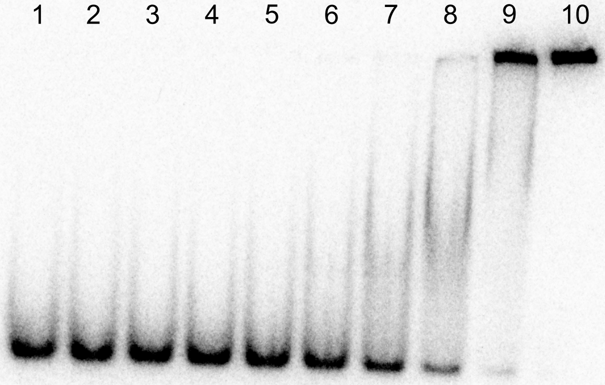

RNA: uncapped
deltaTOP_HsRPS6_Stem

Dr (zebrafish)
DM15 (µM)
1. 0
2. 0.01
3. 0.03
4. 0.1
5. 0.3
6. 1
7. 3
8. 5
9. 10
10. 20

S5 Fig.C middle panel

HsDM15 (μM)
1. 0
2. 0.01
3. 0.03
4. 0.1
5. 0.3
6. 1
7. 3
8. 5
9. 10
10. 20

hDM15 + dTOP_Struc

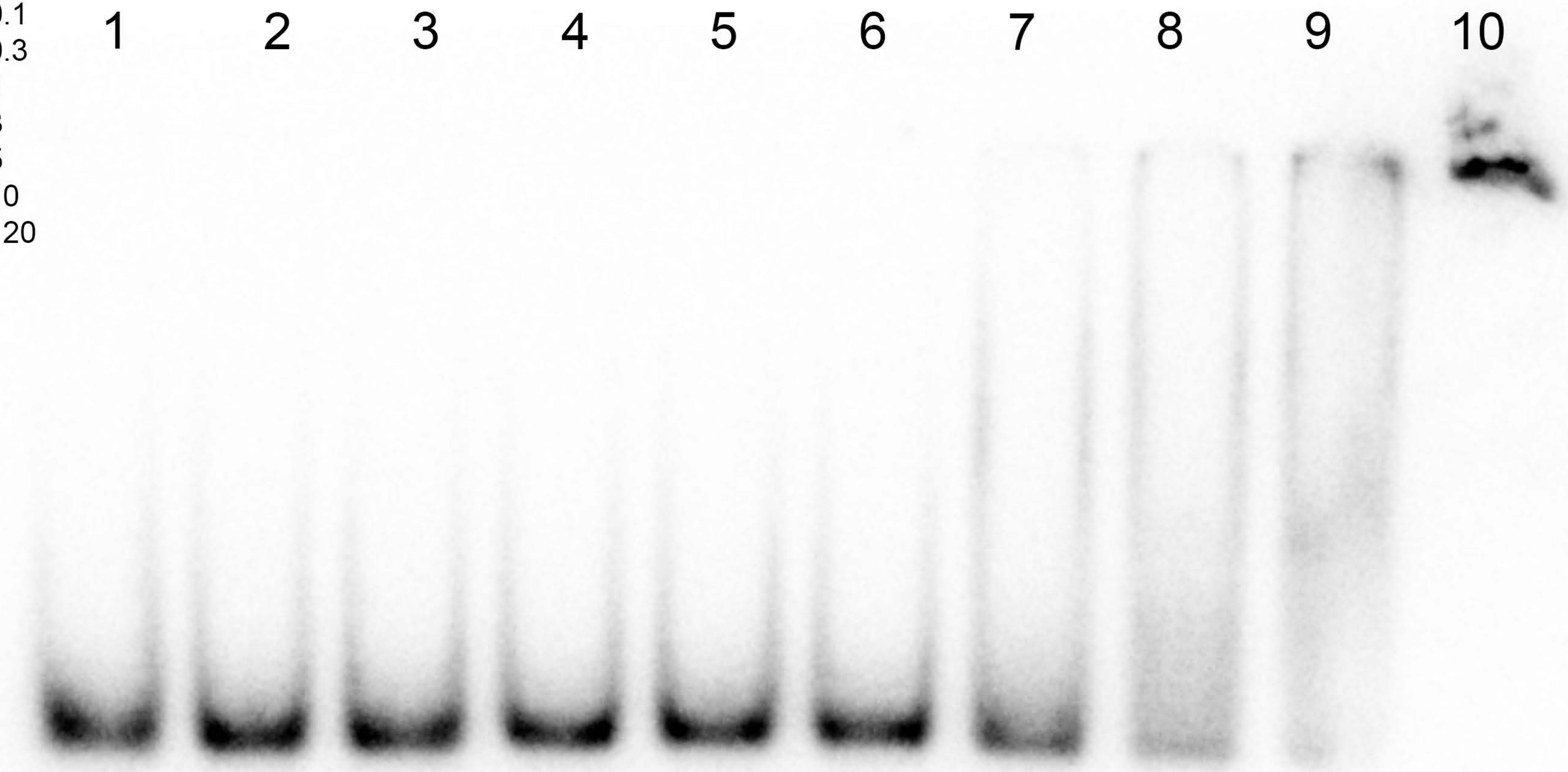

RNA: uncapped
deltaTOP_HsRPS6_Stem

S5 Fig.C bottom panel

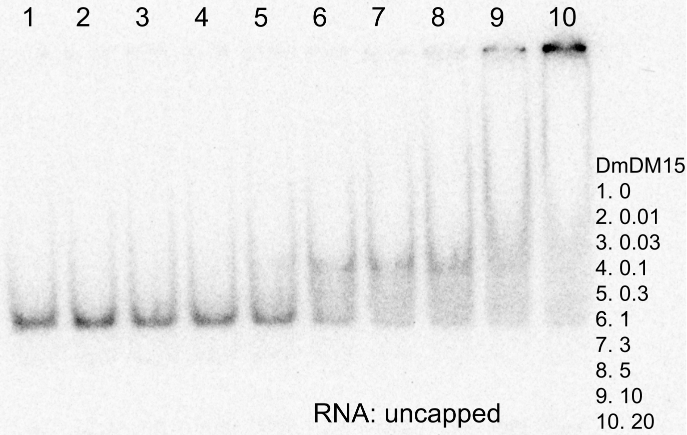

S5 Fig.D top panel

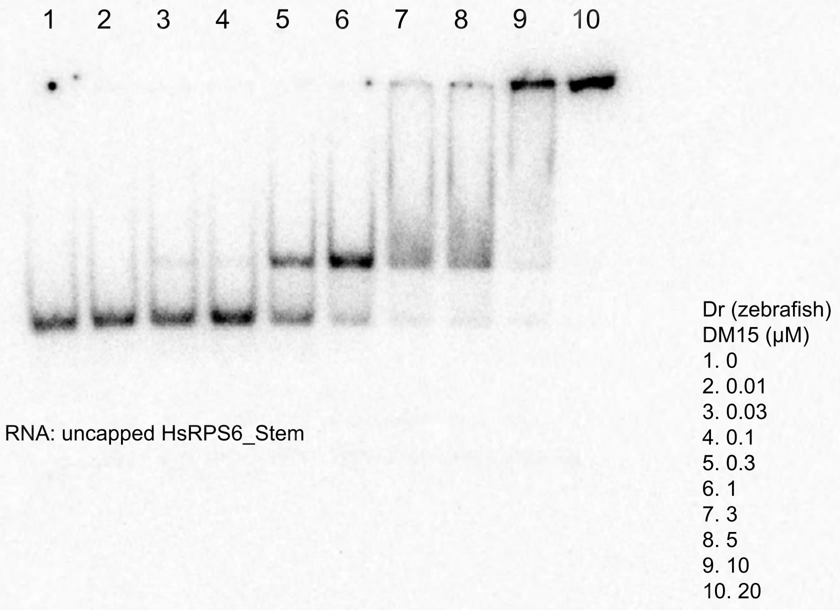

S5 Fig.D middle panel

HsDM15 (μM)
1. 0
2. 0.01
3. 0.03
4. 0.1
5. 0.3
6. 1
7. 3
8. 5
9. 10
10. 20

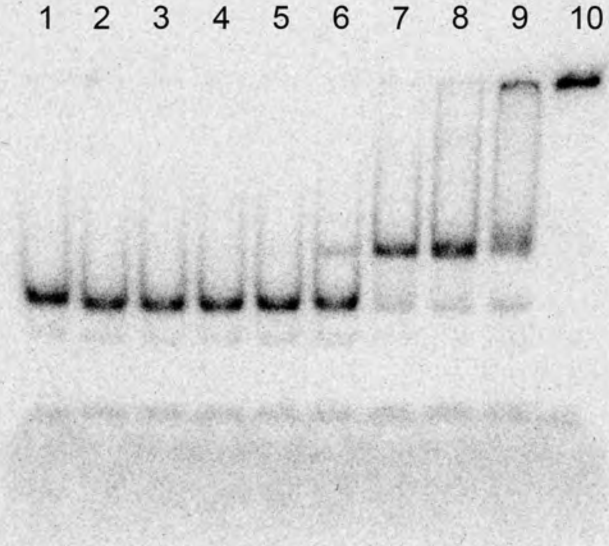

RNA: uncapped HsRPS6_Stem

S5 Fig.D bottom panel

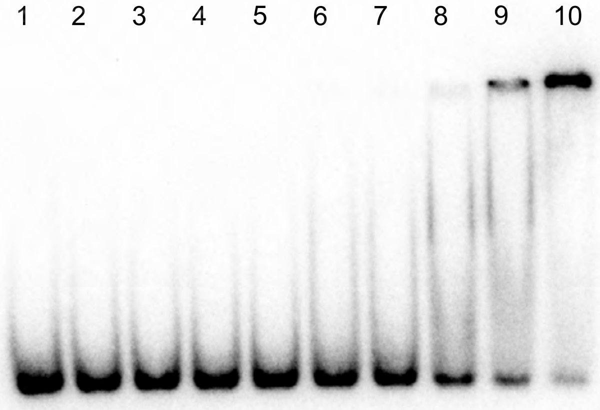

DmDM15 (μM)
1. 0
2. 0.01
3. 0.03
4. 0.1
5. 0.3
6. 1
7. 3
8. 5
9. 10
10. 20

RNA: uncapped
HsPABPC1_Stem

S5 Fig.E top panel

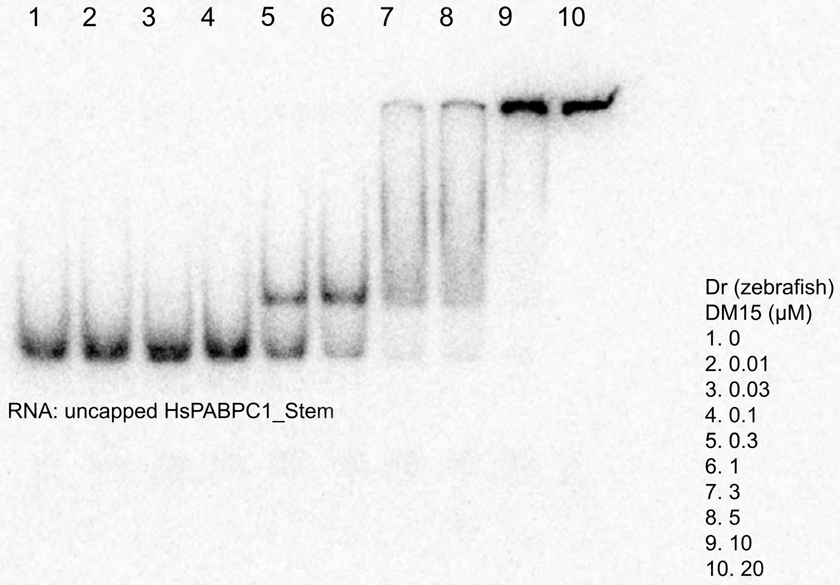

S5 Fig.E middle panel

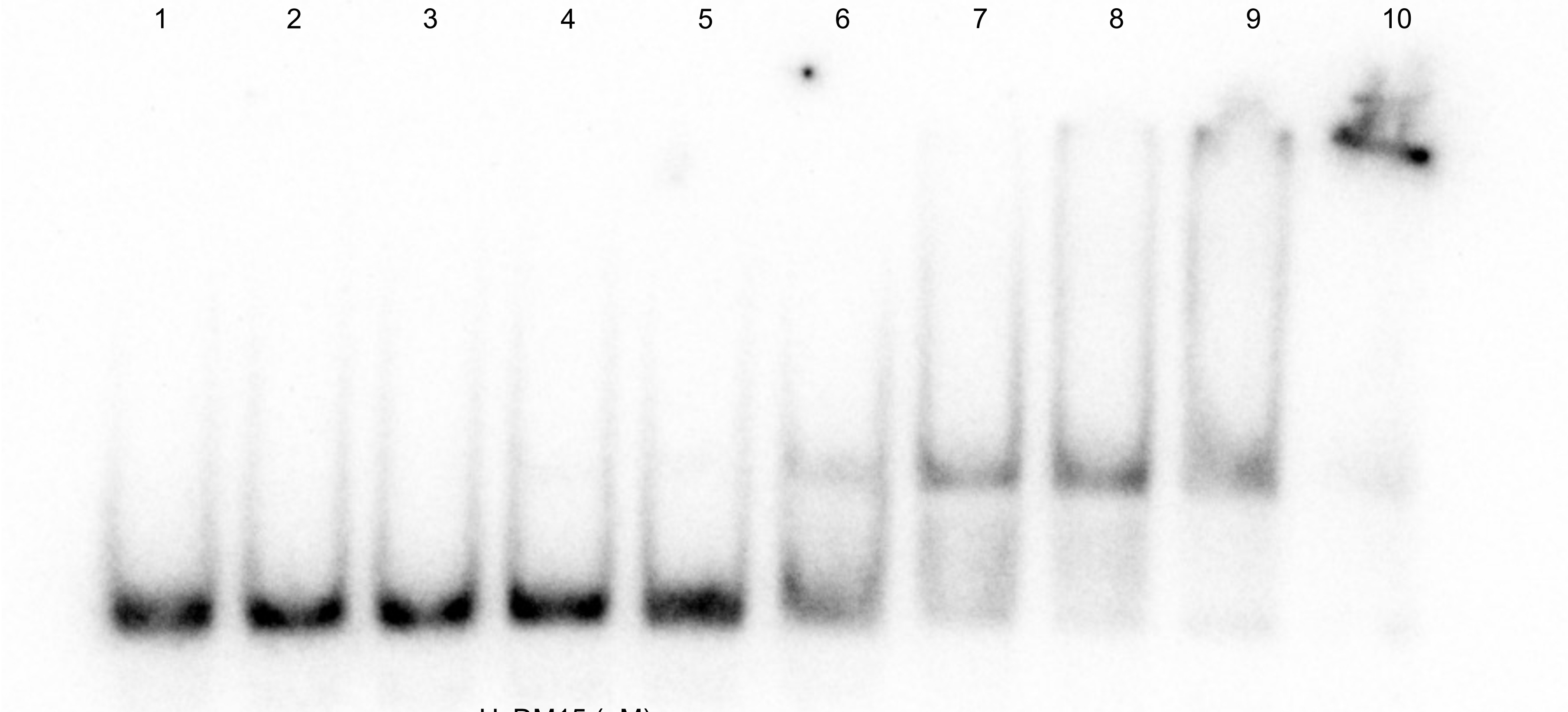

HsDM15 (μM)

1. 0	5. 0.3	9. 10
2. 0.01	6. 1	10. 20
3. 0.03	7. 3	
4. 0.1	8. 5	

Fig S5E bottom panel

RNA: uncapped HsPABPC1_Stem
